# Supplementary material for: Implementation and Evaluation of a Novel Media Education Curriculum for Pediatric Residents
Source: MedEdPORTAL. 2023 Dec 22;19:11372. doi: 10.15766/mep_2374-8265.11372 (PMC10739037; doi:10.15766/mep_2374-8265.11372)
Supplement: Supplementary file 1 — Timeline for Curriculum.docxPretest.docxWorkshop 1 Slides.pptxWorkshop 2 Slides.pptxRole-Play Patient Script.docxRole-Play Physician Guide.docxRole-Play Observation of Performance Checklist.docxPosttest Immediately After Curriculum.docxPosttest 4 Months After Curriculum.docxAnswer Key to Knowledge Questions.docx [file mep_2374-8265.11372-s001.zip › E. Role-Play Patient Script.docx]

**Appendix E: Role Play Patient Script**

**Media Education Workshop #2
Patient Script**

*Use the below responses (with ad-libbing allowed!) during your role-play. If you are asked something that is not in this script, you can make it up.*

**Home**: Lives with mother, father, 2 siblings (10 and 16 y/o). Feel safe and supported at home.

**Education**: Currently 14 years old, in 9th grade at a local public high school.

**Activities**: Love “binge” watching Netflix, spending time on my phone, hanging out with friends.

**Drugs**: Has tried vaping THC before, got it from a friend. Denies drug/alcohol use.

**Sex**: Denies sexual activity. Unsure if interested in females or males.
**Suicidality**: Mood is “up and down”. No suicidal/homicidal ideation.

*If asked* why or when you feel down, it is when you feel lonely like during the pandemic.

*If asked* if you have ways to cope with stress feelings of sadness, you scroll through apps or listen to music. 
*If asked* which apps, you scroll through Instagram and TikTok. You don’t usually post, just like to see what other people are posting.

**Physical Activity**: Ride bike occasionally (*if asked* more specific, 10-15 minutes on weekends)
**Sleep:** School days about 6 hours, weekends about 9-10 hours

**Media** (*only respond if asked*):

- Hours of recreational screen time: During school days maybe 3-4 hours, during weekdays/summer probably 6-8 hours
- Devices in bedroom at night: Yes TV and my phone; on my phone until I fall asleep
- Devices at mealtimes: Yes I pull my phone out of my pocket and my parents don’t care because they’re also watching TV
- Does media affect your sleep: Probably, I stay up late playing video games or scrolling through apps like TikTok
- Does media affect your social life: I don’t think so. I mean yeah sometimes I prefer to be alone with my phone but I still like hanging out with my family.

Does media affect your school performance: No.
