# Supplementary material for: Implementation and Evaluation of a Novel Media Education Curriculum for Pediatric Residents
Source: MedEdPORTAL. 2023 Dec 22;19:11372. doi: 10.15766/mep_2374-8265.11372 (PMC10739037; doi:10.15766/mep_2374-8265.11372)
Supplement: Supplementary file 1 — Timeline for Curriculum.docxPretest.docxWorkshop 1 Slides.pptxWorkshop 2 Slides.pptxRole-Play Patient Script.docxRole-Play Physician Guide.docxRole-Play Observation of Performance Checklist.docxPosttest Immediately After Curriculum.docxPosttest 4 Months After Curriculum.docxAnswer Key to Knowledge Questions.docx [file mep_2374-8265.11372-s001.zip › F. Role-Play Physician Guide.docx]

**Appendix F: Role Play Physician Script**

**Media Education Workshop #2
Physician Script**

*You have been assigned the role of physician for this scenario. Your role play scenario is as follows:*

You are the physician who is performing an annual physical for an adolescent patient. With the patient, you have already completed your introductions, medical history and review of systems. You have learned that this is a 14-year-old patient with no physical complaints. The patient is currently alone in the exam room, as you have asked the parent/guarding to wait in the waiting room.

You are now to perform the HEADSSS exam with the goal of incorporating media-related screening questions and counseling interventions that have been reviewed throughout this curriculum.
