# Supplementary material for: Implementation and Evaluation of a Novel Media Education Curriculum for Pediatric Residents
Source: MedEdPORTAL. 2023 Dec 22;19:11372. doi: 10.15766/mep_2374-8265.11372 (PMC10739037; doi:10.15766/mep_2374-8265.11372)
Supplement: Supplementary file 1 — Timeline for Curriculum.docxPretest.docxWorkshop 1 Slides.pptxWorkshop 2 Slides.pptxRole-Play Patient Script.docxRole-Play Physician Guide.docxRole-Play Observation of Performance Checklist.docxPosttest Immediately After Curriculum.docxPosttest 4 Months After Curriculum.docxAnswer Key to Knowledge Questions.docx [file mep_2374-8265.11372-s001.zip › G. Role-Play Observation of Performance Checklist.docx]

**Appendix G: Role-Play Observation of Performance Checklist**

**Observation of Performance Checklist: Media Role-Play**

*To be filled out by facilitator/observer during role-play scenarios of media education curriculum*

| **Skill Assessed** |  | **Done** | **Done, but in need of remediation** | **Not Done** | **Comment** |
| --- | --- | --- | --- | --- | --- |
| Elicit hours of recreational screen time daily |  |  |  |  |  |
| Elicit types of devices used |  |  |  |  |  |
| Elicit location of devices used |  |  |  |  |  |
| Ask patient how media affects the three S’s | Sleep |  |  |  |  |
|  | School performance |  |  |  |  |
|  | Social life |  |  |  |  |
| Counsel regarding limiting hours of recreational screen time to 2 hours or less |  |  |  |  |  |
| Counsel regarding location of device charging overnight (not in bedroom) |  |  |  |  |  |
| Counsel regarding avoiding screen use 1 hour before bedtime |  |  |  |  |  |
| Counsel regarding avoiding screen use during mealtimes |  |  |  |  |  |
| Provide resources to patient/family about media use | Examples: AAP Family Media Use Plan, CommonSenseMedia.org, or Digital Wellness Lab |  |  |  |  |
