# Supplementary material for: Implementation and Evaluation of a Novel Media Education Curriculum for Pediatric Residents
Source: MedEdPORTAL. 2023 Dec 22;19:11372. doi: 10.15766/mep_2374-8265.11372 (PMC10739037; doi:10.15766/mep_2374-8265.11372)
Supplement: Supplementary file 1 — Timeline for Curriculum.docxPretest.docxWorkshop 1 Slides.pptxWorkshop 2 Slides.pptxRole-Play Patient Script.docxRole-Play Physician Guide.docxRole-Play Observation of Performance Checklist.docxPosttest Immediately After Curriculum.docxPosttest 4 Months After Curriculum.docxAnswer Key to Knowledge Questions.docx [file mep_2374-8265.11372-s001.zip › I. Posttest 4 Months After Curriculum.docx]

**Appendix I: Post-Test (4 Months After Curriculum)**

**Media Education Post-Test (4 months later)**

*Please answer the below questions to the best of your ability without using any resources.*

1. Which year do you graduate pediatric residency?

- 2022
- 2023
- 2024

1. Select your gender:

- Male
- Female
- Non-binary / third gender
- Prefer not to say

1. What are your plans post-residency?

- General Pediatrics Practice
- Pediatric Subspecialty Fellowship
- Non-pediatric residency or fellowship
- Chief Residency
- Other position
- No job or position at this time
- Undecided

1. Prior to residency, did you receive any education on health effects of media use, how to screen patients for media use, or how to counsel patients regarding media use?

- Yes
- No
- I don’t know

1. Which of the following IS a studied benefit of broadcast media and/or social media on children and adolescents, with regard to personal development, as established by the American Academy of Pediatrics (AAP)? Select ONE answer.

- Identification of role models
- Enhance wellness and promote healthy behaviors
- Platform to showcase talents
- Development of personal identity

1. Which of the following is NOT a studied benefit of broadcast media and/or social media on children and adolescents, with regard to social behaviors, as established by the AAP? Select ONE answer.

- Virtual collaboration with students on assignments
- Communication with family and friends who are geographically far
- Gain followers and praise on social media
- Promotion of community participation and civic engagement
- Social inclusion among those who otherwise feel excluded

1. Which of the following is NOT a studied benefit of broadcast media and/or social media on children and adolescents, with regard to knowledge of new information, as established by the AAP? Select ONE answer.

- Raise awareness of current events
- Exposure to new ideas/information
- Understanding of adolescent behaviors from media portrayals of adolescents
- Supplementary source of health information to healthcare visits

1. Which of the following is NOT a studied risk of broadcast media and/or social media on children and adolescents, with regard to social behaviors, as established by the AAP? Select ONE answer.

- Distraction from community participation and civic engagement
- Decreased parental engagement with children
- Cyberbullying
- Sexting or exploitation of children by sex offenders
- Decreased interest in “real life” relationships

1. Which of the following is NOT a studied risk of broadcast media and/or social media on children and adolescents, with regard to physical or mental health, as established by the AAP? Select ONE answer.

- Obesity
- Sleep disturbances
- Earlier initiation of risky behaviors including substance use, sexual behaviors, self-injury, disordered eating
- Increased risk of depression
- Addiction to technology

1. How many hours of sedentary screen time should children (aged 2 through 18 years of age) have daily based on AAP recommendations?

- 0 hours
- 1 hour or less
- 2 hours or less
- 3 hours or less
- 5 hours or less

1. How often, in the past 4 months, have you screened a child/adolescent during a well child visit for the following? Place an X in the appropriate box for each question:

|  | Never | Sometimes | About half the time | Most of the time | Always |
| --- | --- | --- | --- | --- | --- |
| Hours of recreational screen time |  |  |  |  |  |
| Types of media being used (e.g. TV shows, videogames, social media networks, etc.) |  |  |  |  |  |
| Location of media devices in house (e.g. bedroom, living room, kitchen, etc.) |  |  |  |  |  |
| Times that media is being used (bedtime, mealtimes, etc.) |  |  |  |  |  |
| How media affects patient’s sleep |  |  |  |  |  |
| How media affects patient’s school performance |  |  |  |  |  |
| How media affects patient’s social life |  |  |  |  |  |

1. How often, in the past 4 months, have you counseled a patient/family during a **well child visit**on the following? Place an X in the appropriate box for each question:

|  | Never | Sometimes | About half the time | Most of the time | Always |
| --- | --- | --- | --- | --- | --- |
| Limit recreational screen time to 2 hours or less |  |  |  |  |  |
| Remove devices from bedroom and/or dining room table |  |  |  |  |  |
| Avoid screen use 1 hour before bedtime |  |  |  |  |  |

1. How often, in the past four months, have you provided resources regarding media use to a patient/family (i.e. AAP Family Media Use Plan, patient materials from DigitalWellnessLab.org, or CommonSenseMedia.org)?

- 0 times
- 1-2 times
- 3-4 times
- More than 4 times

1. Please select how strongly you agree with each of the following statements.

|  | Strongly Disagree | Disagree | Neither agree nor disagree | Agree | Strongly Agree |
| --- | --- | --- | --- | --- | --- |
| I believe media use is a health issue for children and adolescents. |  |  |  |  |  |
| I believe that my residency program adequately prepares residents to screen for media use during well child visits. |  |  |  |  |  |
| I believe that my residency program adequately prepares residents to provide counseling about media use during well child visits. |  |  |  |  |  |
| I feel confident screening a patient for media use. |  |  |  |  |  |
| I feel confident counseling a patient/family on media use. |  |  |  |  |  |
| I believe that media counseling by pediatricians during all well child visits is necessary and important. |  |  |  |  |  |
| I feel that this media education curriculum is relevant to my job. |  |  |  |  |  |
| I feel that this media education curriculum was engaging. |  |  |  |  |  |
| I feel that this media education curriculum should be provided to all pediatric residents. |  |  |  |  |  |
