# Supplementary material for: Implementation and Evaluation of a Novel Media Education Curriculum for Pediatric Residents
Source: MedEdPORTAL. 2023 Dec 22;19:11372. doi: 10.15766/mep_2374-8265.11372 (PMC10739037; doi:10.15766/mep_2374-8265.11372)
Supplement: Supplementary file 1 — Timeline for Curriculum.docxPretest.docxWorkshop 1 Slides.pptxWorkshop 2 Slides.pptxRole-Play Patient Script.docxRole-Play Physician Guide.docxRole-Play Observation of Performance Checklist.docxPosttest Immediately After Curriculum.docxPosttest 4 Months After Curriculum.docxAnswer Key to Knowledge Questions.docx [file mep_2374-8265.11372-s001.zip › J. Answer Key to Knowledge Questions.docx]

**Appendix J: Answer Key to Questions #5-10 of Pre- and Post-Tests**

Media Education: Pre-Test & Post-Test Answer Key

5. Which of the following IS a studied benefit of broadcast media and/or social media on children and adolescents, with regard to personal development, as established by the American Academy of Pediatrics (AAP)? Select ONE right answer.

- Identification of role models
- Enhance wellness and promote healthy behaviors
- Platform to showcase talents
- Development of personal identity

1. Which of the following is NOT a studied benefit of broadcast media and/or social media on children and adolescents, with regard to social behaviors, as established by the AAP? Select ONE right answer.

- Virtual collaboration with students on assignments
- Communication with family and friends who are geographically far
- Gain followers and praise on social media
- Promotion of community participation and civic engagement
- Social inclusion among those who otherwise feel excluded

1. Which of the following is NOT a studied benefit of broadcast media and/or social media on children and adolescents, with regard to knowledge of new information, as established by the AAP?

- Raise awareness of current events
- Exposure to new ideas/information
- Understanding of adolescent behaviors from media portrayals of adolescents
- Supplementary source of health information to healthcare visits

1. Which of the following is NOT a studied risk of broadcast media and/or social media on children and adolescents, with regard to social behaviors, as established by the AAP?

- Distraction from community participation and civic engagement
- Decreased parental engagement with children
- Cyberbullying
- Sexting or exploitation of children by sex offenders
- Decreased interest in “real life” relationships

1. Which of the following is NOT a studied risk of broadcast media and/or social media on children and adolescents, with regard to physical or mental health, as established by the AAP?

- Obesity
- Sleep disturbances
- Earlier initiation of risky behaviors including substance use, sexual behaviors, self-injury, disordered eating
- Increased risk of depression
- Addiction to technology

1. How many hours of sedentary screen time should children (aged 2 through 18 years of age) have daily based on AAP recommendations?

- 0 hours
- 1 hour or less
- 2 hours or less
- 3 hours or less
- 5 hours or less
