# Supplementary material for: Implementation and Evaluation of a Novel Media Education Curriculum for Pediatric Residents
Source: MedEdPORTAL. 2023 Dec 22;19:11372. doi: 10.15766/mep_2374-8265.11372 (PMC10739037; doi:10.15766/mep_2374-8265.11372)
Supplement: Supplementary file 1 — Timeline for Curriculum.docxPretest.docxWorkshop 1 Slides.pptxWorkshop 2 Slides.pptxRole-Play Patient Script.docxRole-Play Physician Guide.docxRole-Play Observation of Performance Checklist.docxPosttest Immediately After Curriculum.docxPosttest 4 Months After Curriculum.docxAnswer Key to Knowledge Questions.docx [file mep_2374-8265.11372-s001.zip › C. Workshop 1 Slides.pptx]

## Slide 1
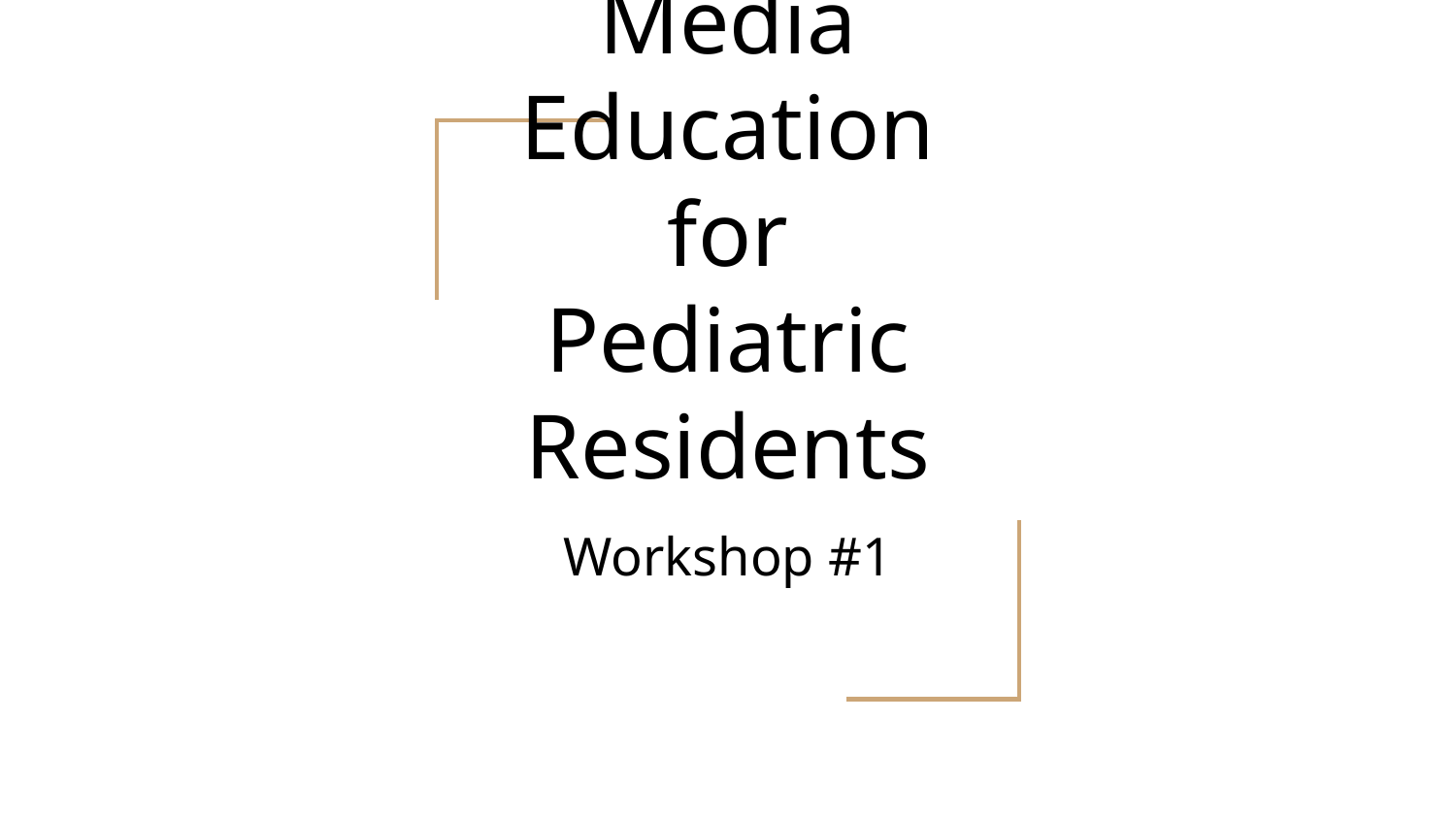

# Media Education for Pediatric Residents
Workshop #1

## Slide 2
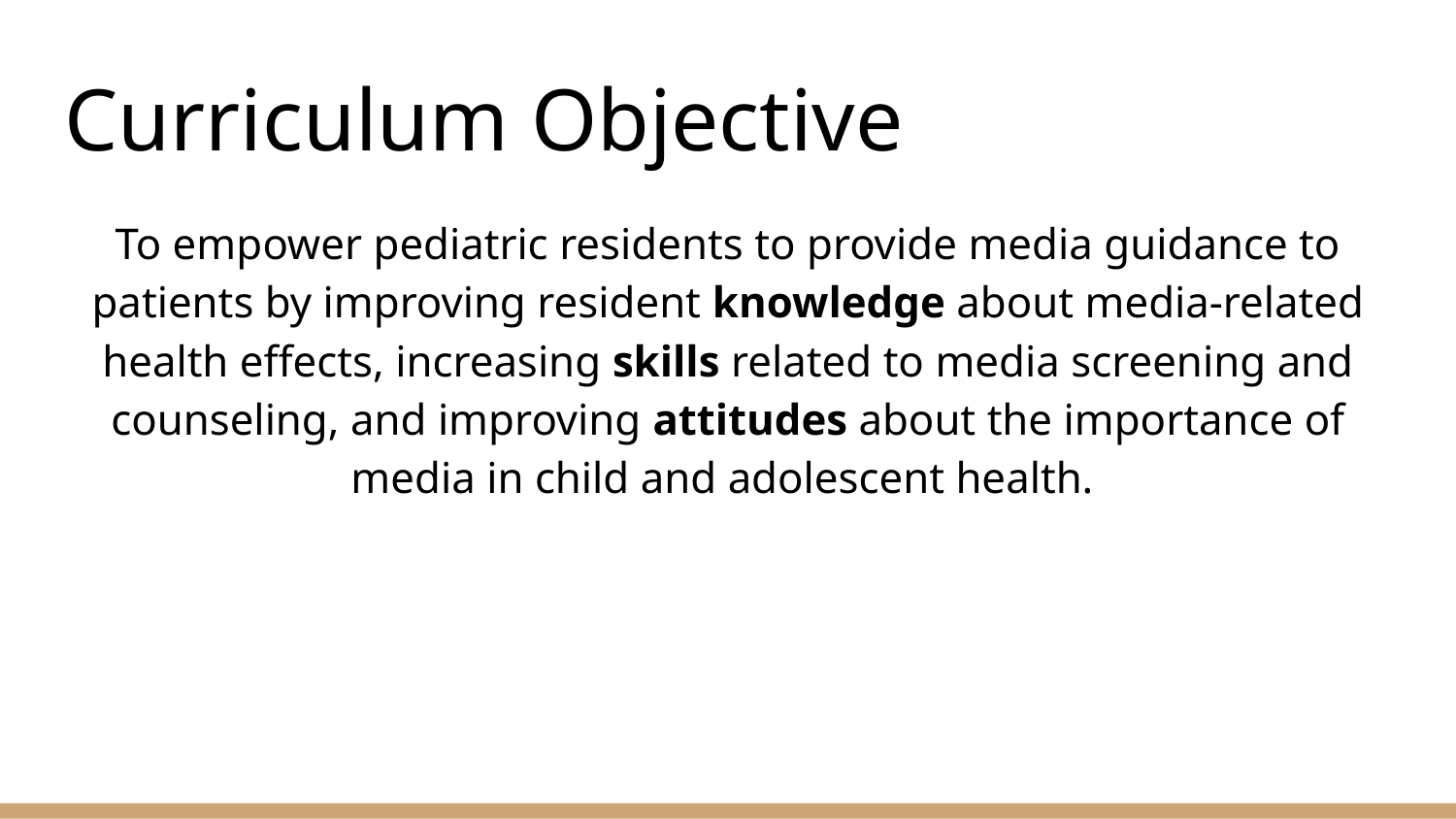

# Curriculum Objective
To empower pediatric residents to provide media guidance to patients by improving resident knowledge about media-related health effects, increasing skills related to media screening and counseling, and improving attitudes about the importance of media in child and adolescent health.

## Slide 3
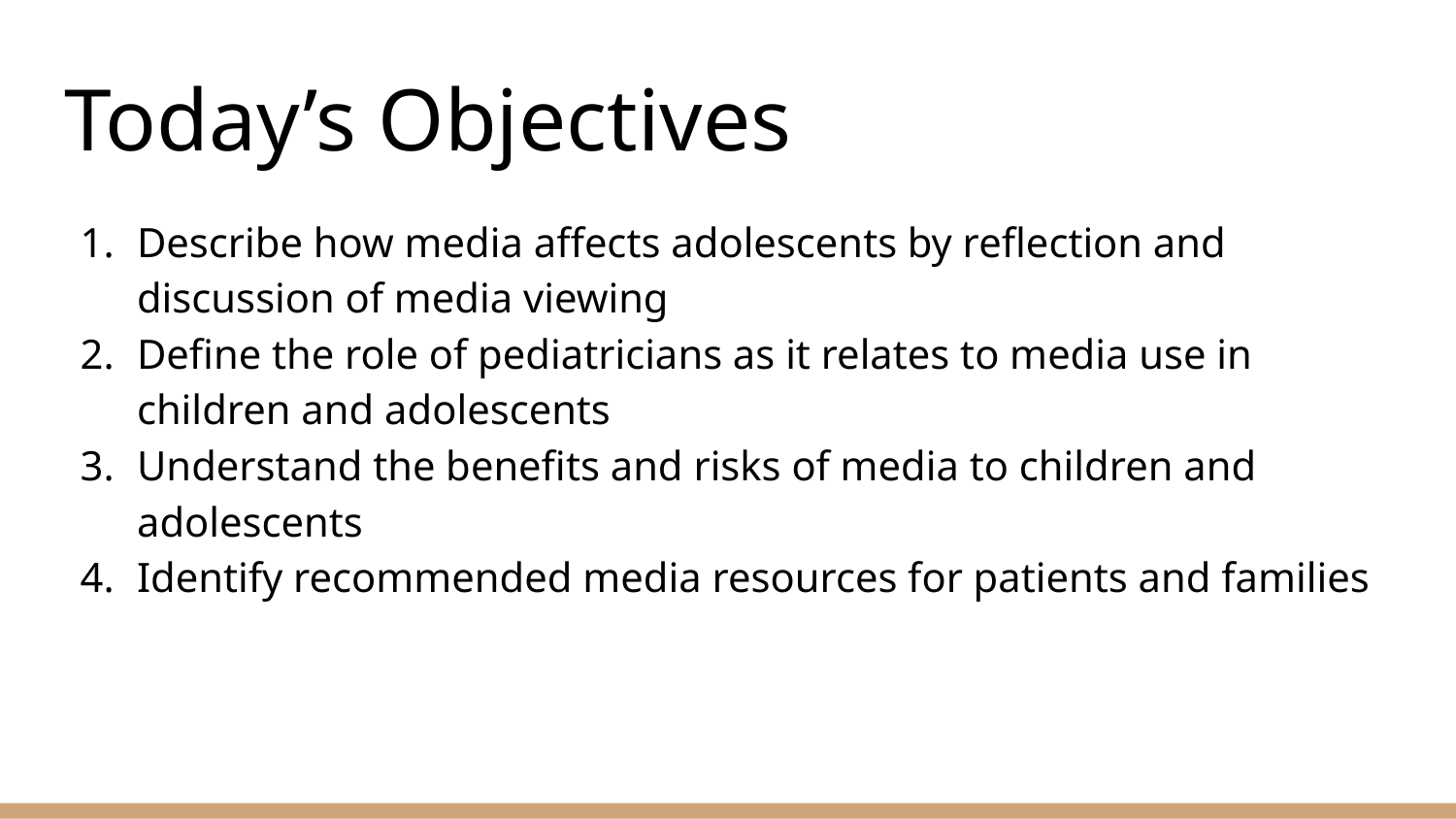

# Today’s Objectives
Describe how media affects adolescents by reflection and discussion of media viewing
Define the role of pediatricians as it relates to media use in children and adolescents
Understand the benefits and risks of media to children and adolescents
Identify recommended media resources for patients and families

## Slide 4
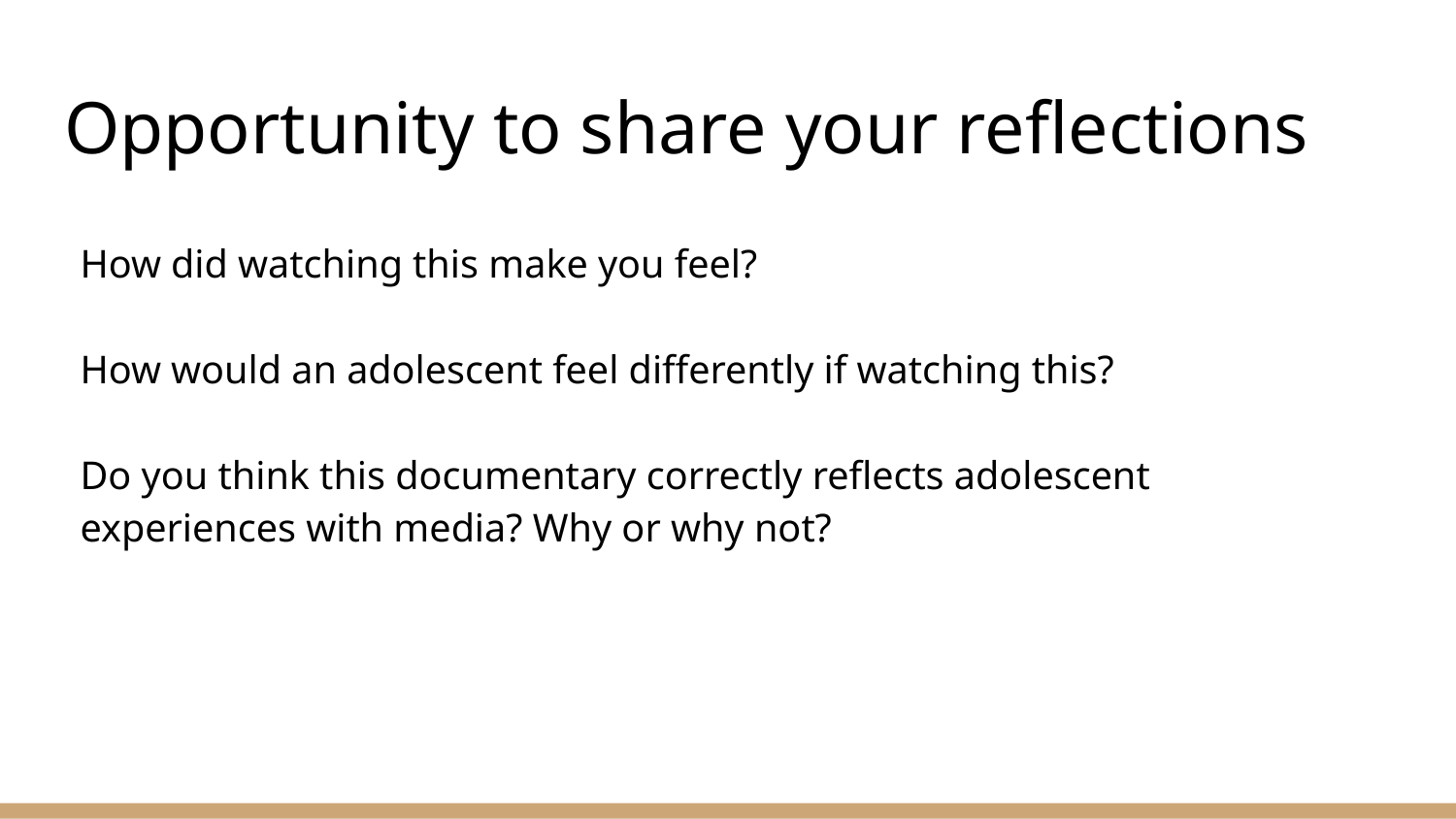

# Opportunity to share your reflections
How did watching this make you feel?
How would an adolescent feel differently if watching this?
Do you think this documentary correctly reflects adolescent experiences with media? Why or why not?

## Slide 5
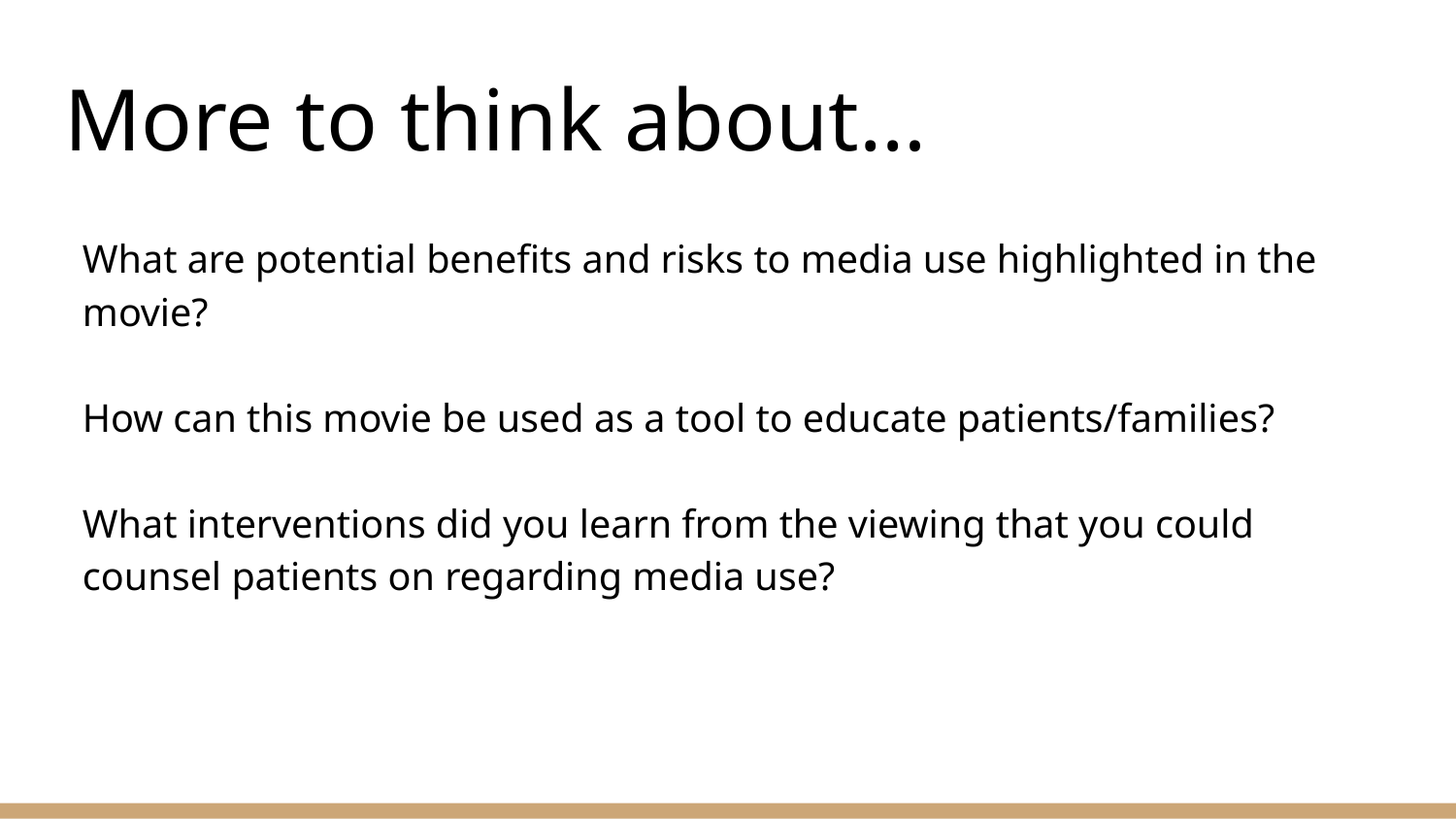

# More to think about…
What are potential benefits and risks to media use highlighted in the movie?
How can this movie be used as a tool to educate patients/families?
What interventions did you learn from the viewing that you could counsel patients on regarding media use?

## Slide 6
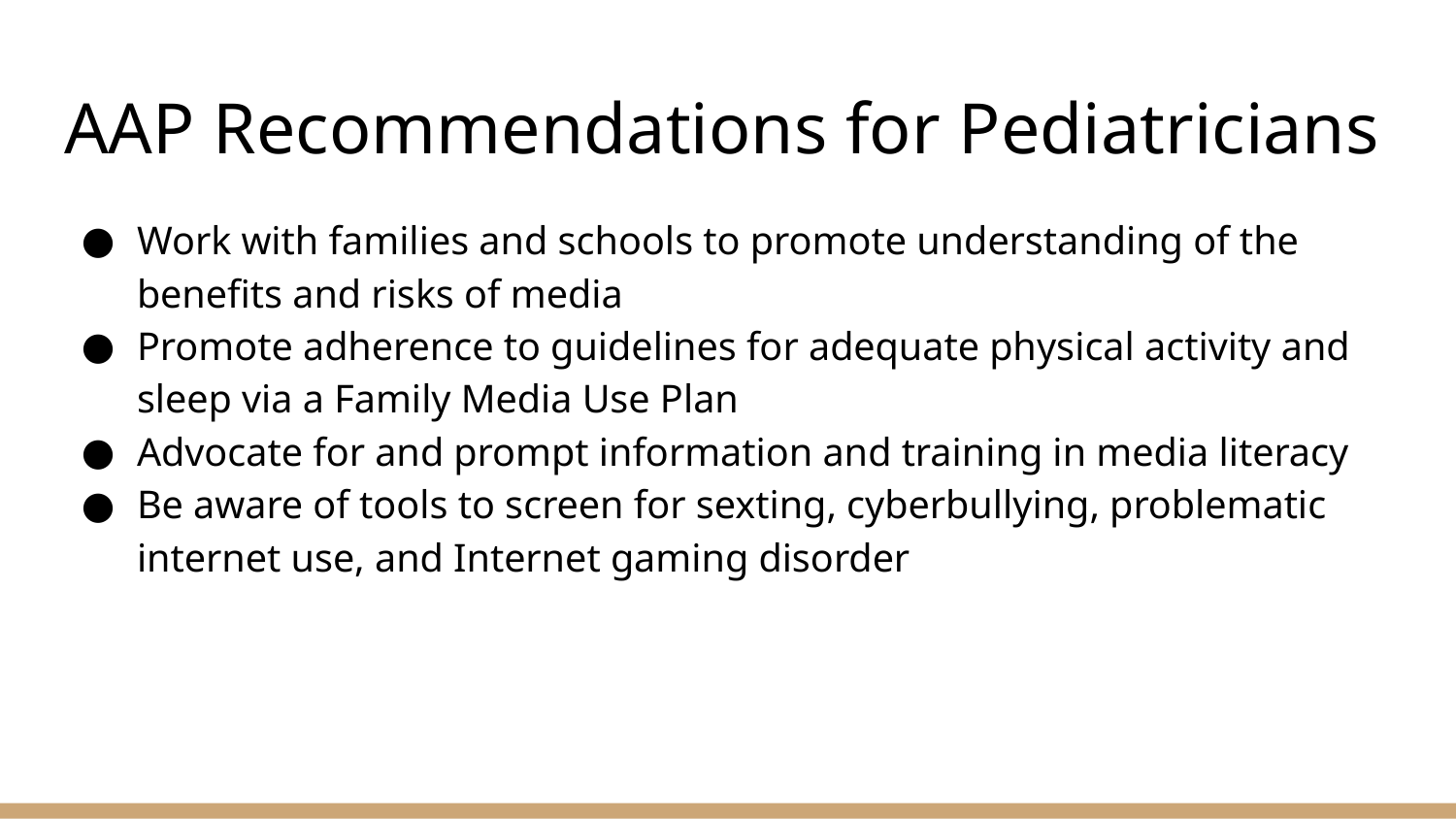

# AAP Recommendations for Pediatricians
Work with families and schools to promote understanding of the benefits and risks of media
Promote adherence to guidelines for adequate physical activity and sleep via a Family Media Use Plan
Advocate for and prompt information and training in media literacy
Be aware of tools to screen for sexting, cyberbullying, problematic internet use, and Internet gaming disorder

## Slide 7
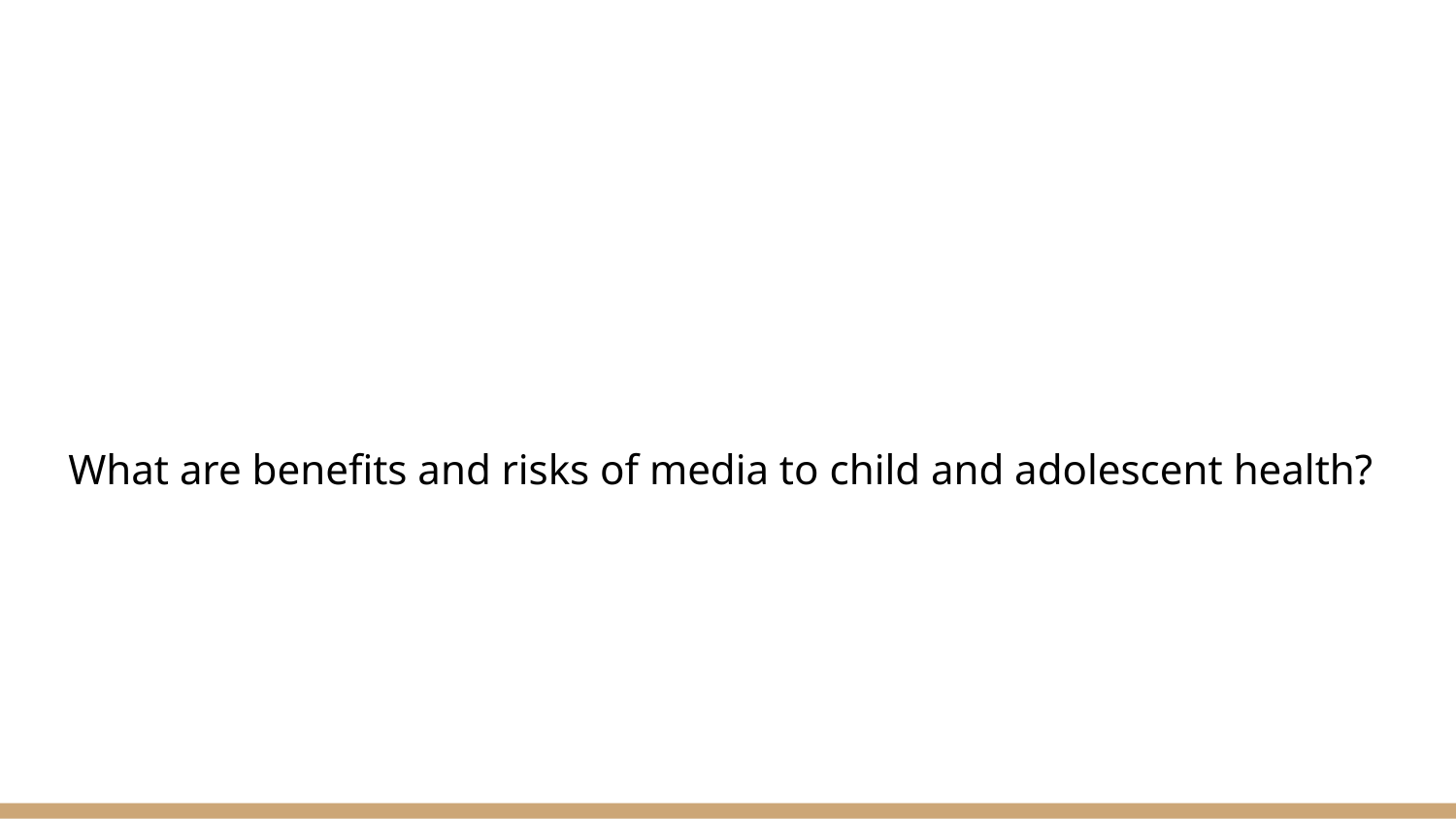

# What are benefits and risks of media to child and adolescent health?

## Slide 8
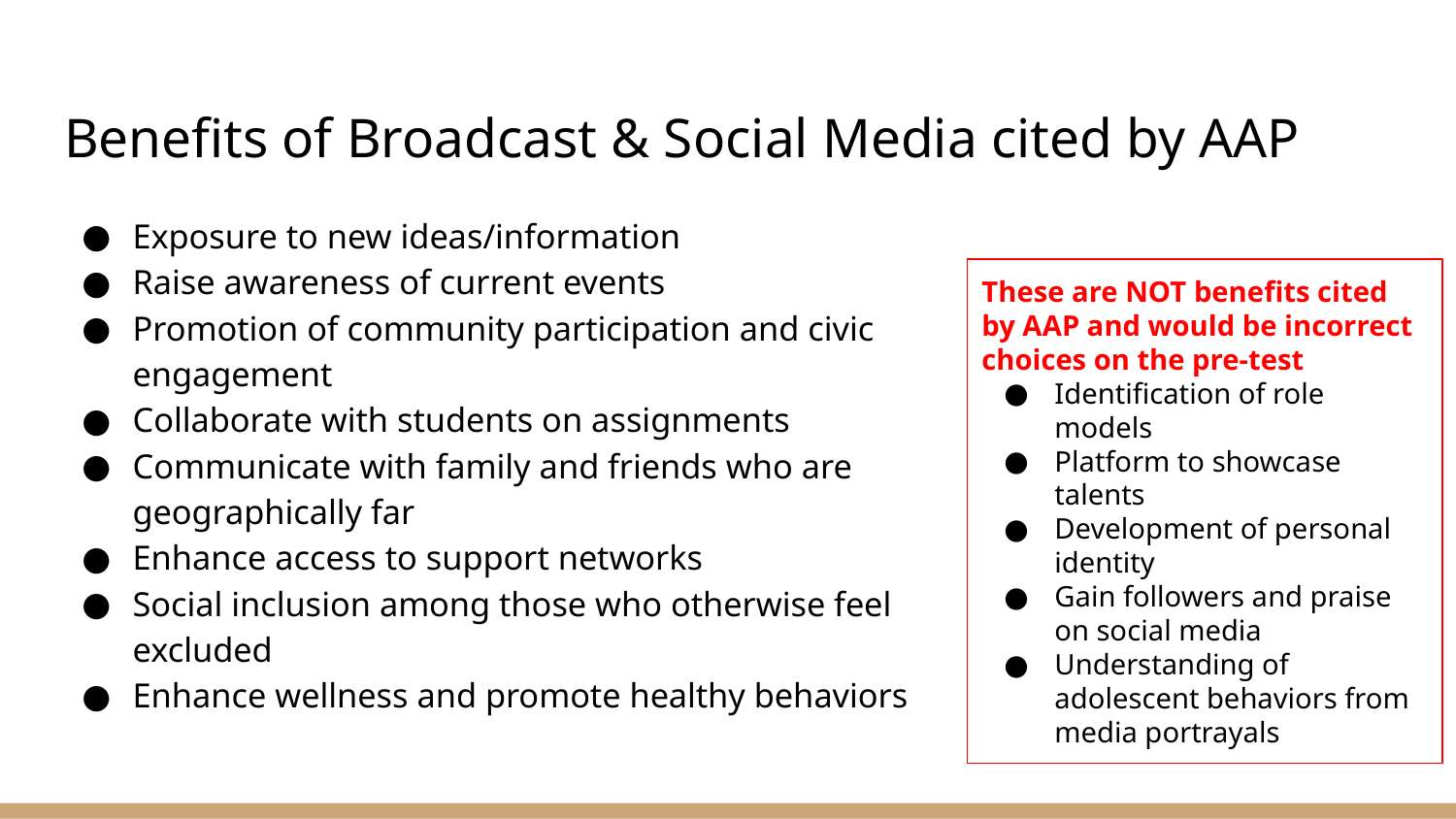

# Benefits of Broadcast & Social Media cited by AAP
Exposure to new ideas/information
Raise awareness of current events
Promotion of community participation and civic engagement
Collaborate with students on assignments
Communicate with family and friends who are geographically far
Enhance access to support networks
Social inclusion among those who otherwise feel excluded
Enhance wellness and promote healthy behaviors
These are NOT benefits cited by AAP and would be incorrect choices on the pre-test
Identification of role models
Platform to showcase talents
Development of personal identity
Gain followers and praise on social media
Understanding of adolescent behaviors from media portrayals

## Slide 9
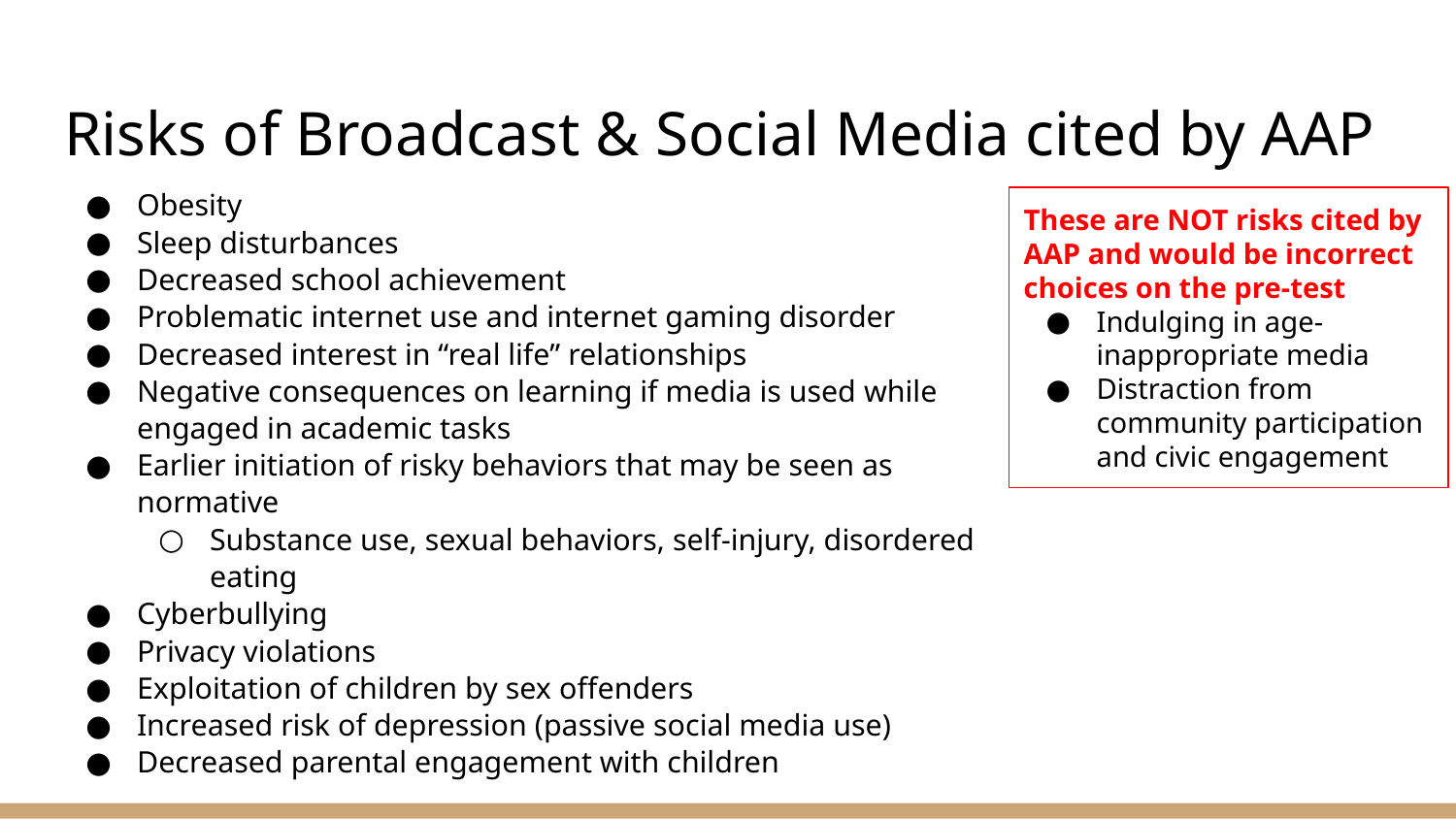

# Risks of Broadcast & Social Media cited by AAP
Obesity
Sleep disturbances
Decreased school achievement
Problematic internet use and internet gaming disorder
Decreased interest in “real life” relationships
Negative consequences on learning if media is used while engaged in academic tasks
Earlier initiation of risky behaviors that may be seen as normative
Substance use, sexual behaviors, self-injury, disordered eating
Cyberbullying
Privacy violations
Exploitation of children by sex offenders
Increased risk of depression (passive social media use)
Decreased parental engagement with children
These are NOT risks cited by AAP and would be incorrect choices on the pre-test
Indulging in age-inappropriate media
Distraction from community participation and civic engagement

## Slide 10
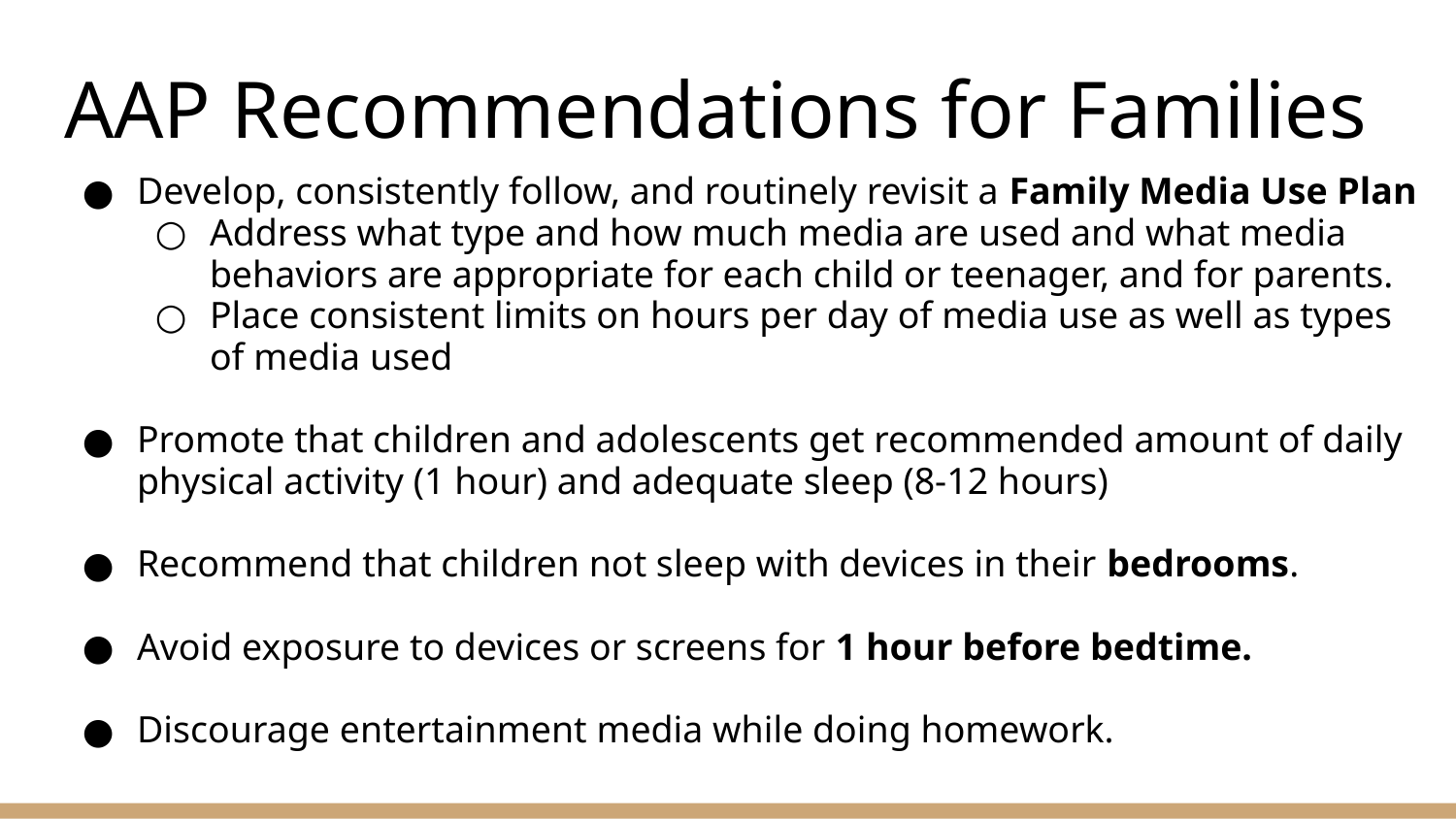

# AAP Recommendations for Families
Develop, consistently follow, and routinely revisit a Family Media Use Plan
Address what type and how much media are used and what media behaviors are appropriate for each child or teenager, and for parents.
Place consistent limits on hours per day of media use as well as types of media used
Promote that children and adolescents get recommended amount of daily physical activity (1 hour) and adequate sleep (8-12 hours)
Recommend that children not sleep with devices in their bedrooms.
Avoid exposure to devices or screens for 1 hour before bedtime.
Discourage entertainment media while doing homework.

## Slide 11
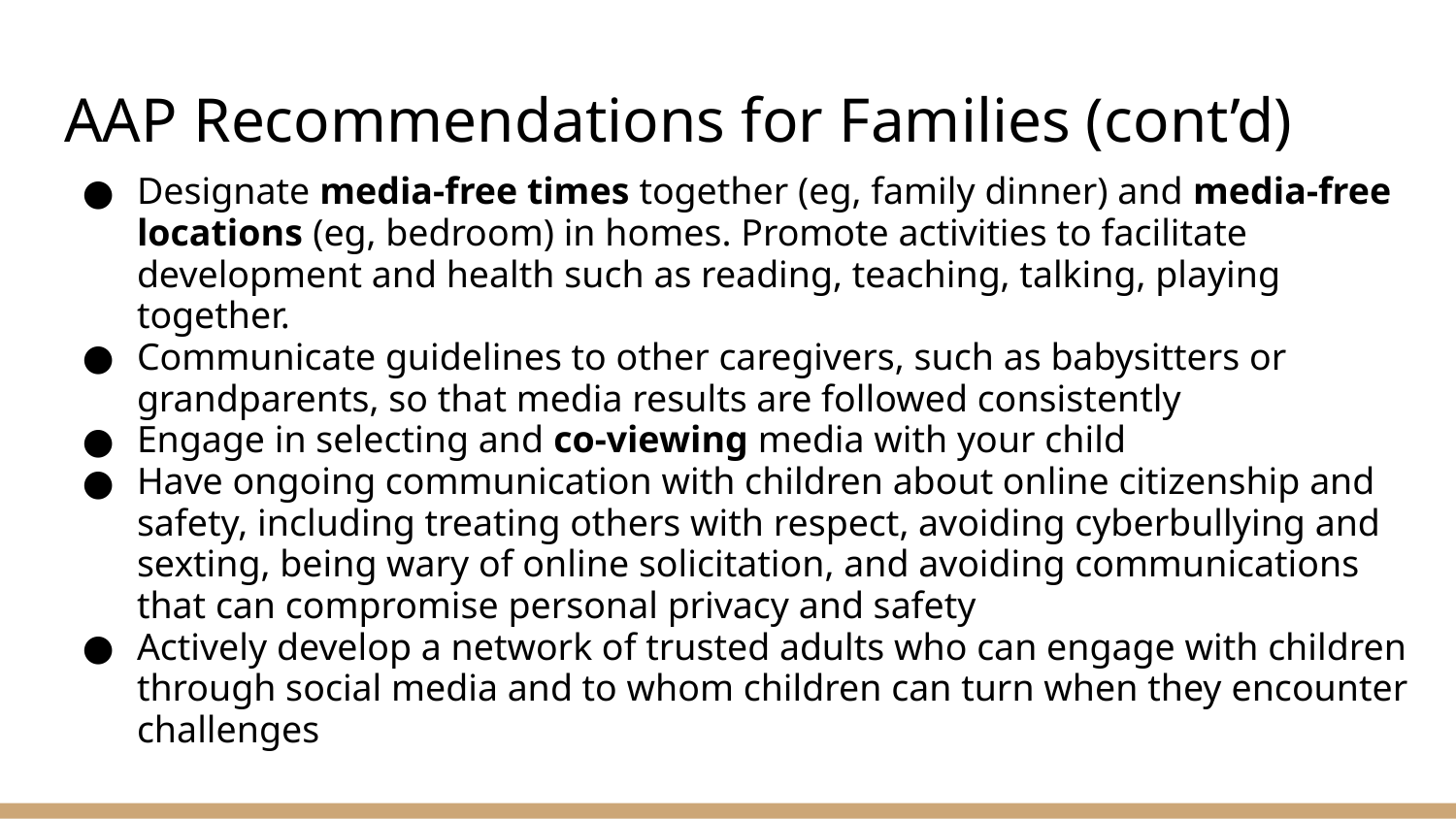

# AAP Recommendations for Families (cont’d)
Designate media-free times together (eg, family dinner) and media-free locations (eg, bedroom) in homes. Promote activities to facilitate development and health such as reading, teaching, talking, playing together.
Communicate guidelines to other caregivers, such as babysitters or grandparents, so that media results are followed consistently
Engage in selecting and co-viewing media with your child
Have ongoing communication with children about online citizenship and safety, including treating others with respect, avoiding cyberbullying and sexting, being wary of online solicitation, and avoiding communications that can compromise personal privacy and safety
Actively develop a network of trusted adults who can engage with children through social media and to whom children can turn when they encounter challenges

## Slide 12
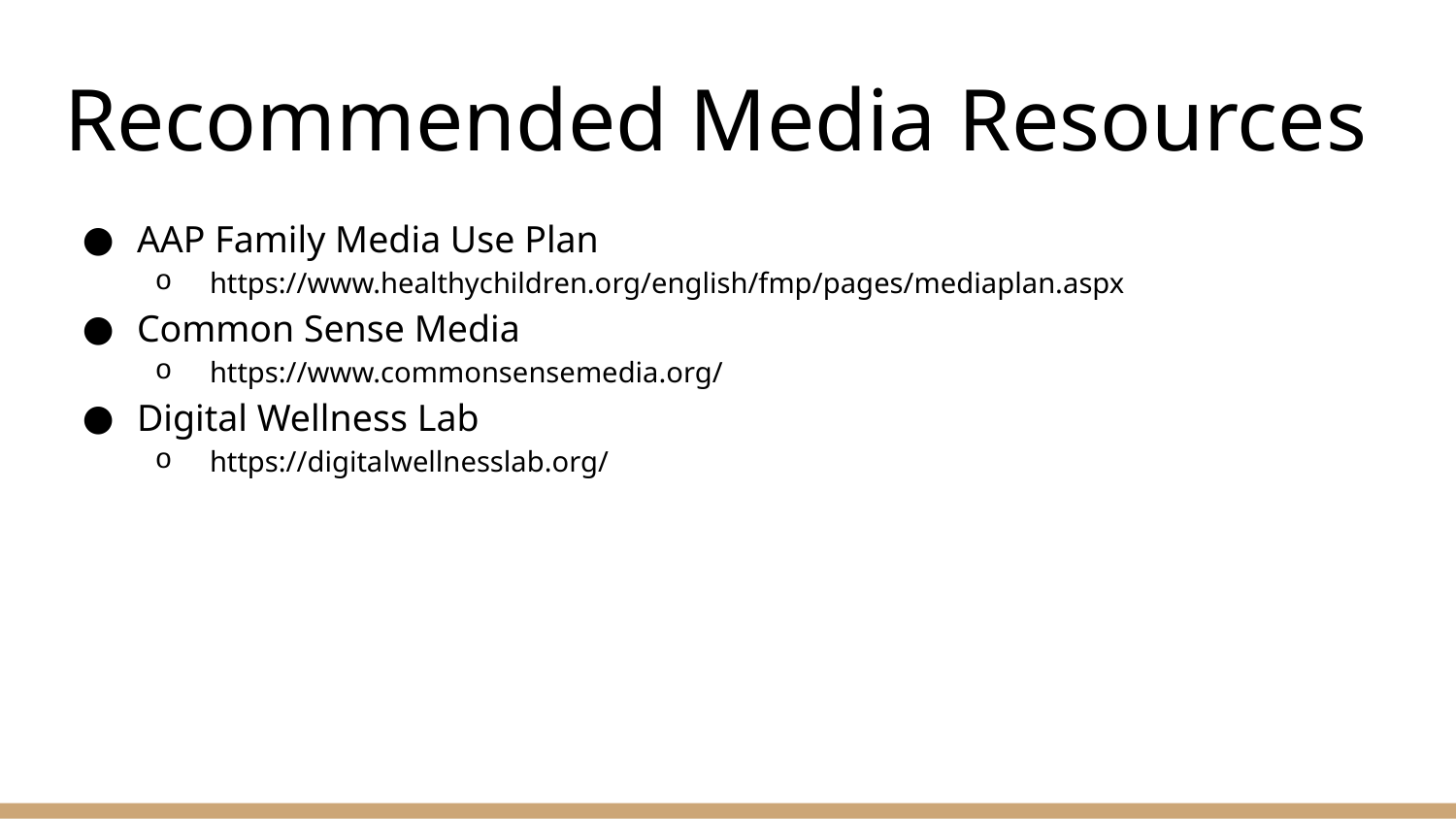

# Recommended Media Resources
AAP Family Media Use Plan
https://www.healthychildren.org/english/fmp/pages/mediaplan.aspx
Common Sense Media
https://www.commonsensemedia.org/
Digital Wellness Lab
https://digitalwellnesslab.org/

## Slide 13
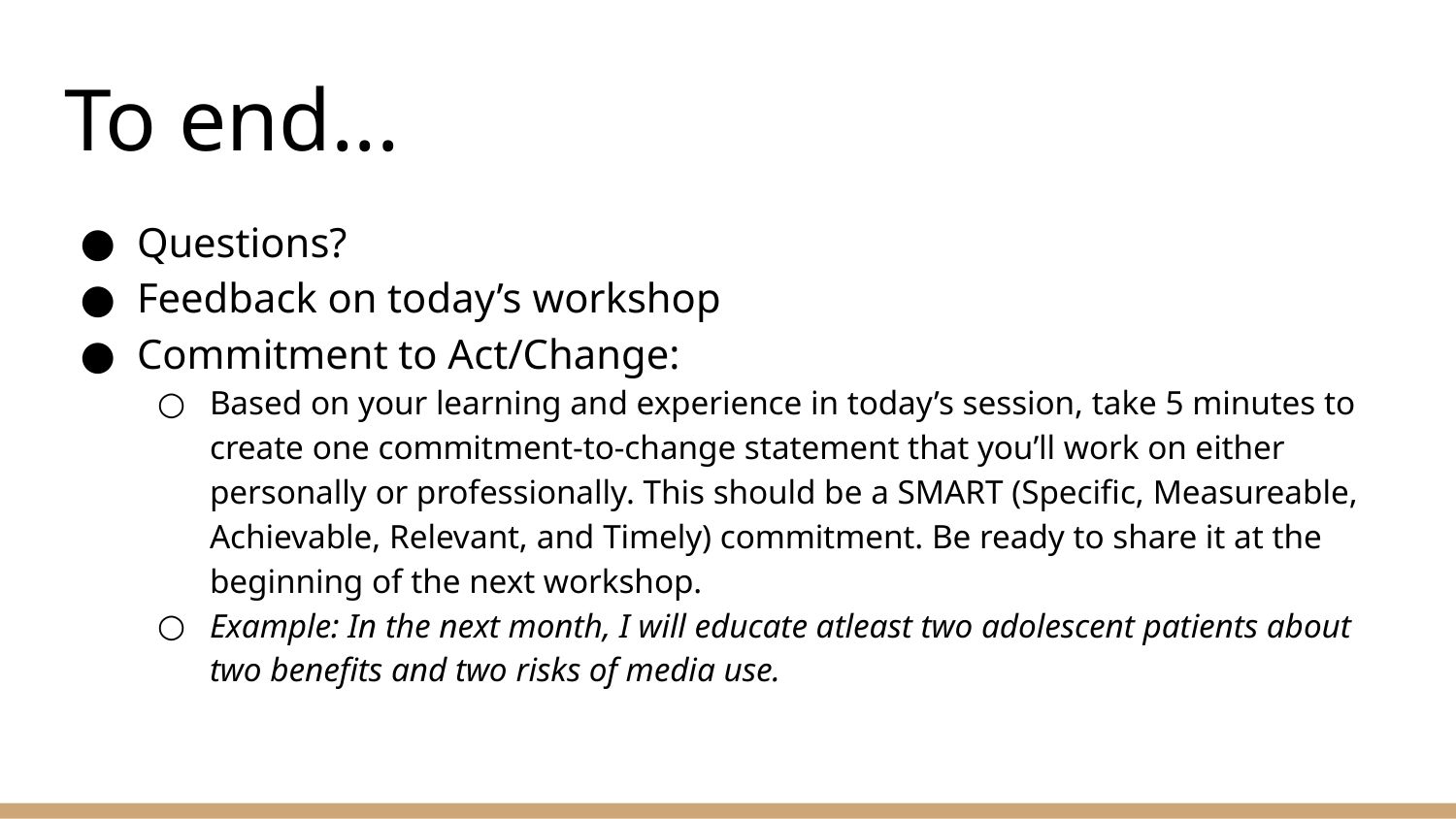

# To end...
Questions?
Feedback on today’s workshop
Commitment to Act/Change:
Based on your learning and experience in today’s session, take 5 minutes to create one commitment-to-change statement that you’ll work on either personally or professionally. This should be a SMART (Specific, Measureable, Achievable, Relevant, and Timely) commitment. Be ready to share it at the beginning of the next workshop.
Example: In the next month, I will educate atleast two adolescent patients about two benefits and two risks of media use.
