# Supplementary material for: Implementation and Evaluation of a Novel Media Education Curriculum for Pediatric Residents
Source: MedEdPORTAL. 2023 Dec 22;19:11372. doi: 10.15766/mep_2374-8265.11372 (PMC10739037; doi:10.15766/mep_2374-8265.11372)
Supplement: Supplementary file 1 — Timeline for Curriculum.docxPretest.docxWorkshop 1 Slides.pptxWorkshop 2 Slides.pptxRole-Play Patient Script.docxRole-Play Physician Guide.docxRole-Play Observation of Performance Checklist.docxPosttest Immediately After Curriculum.docxPosttest 4 Months After Curriculum.docxAnswer Key to Knowledge Questions.docx [file mep_2374-8265.11372-s001.zip › D. Workshop 2 Slides.pptx]

## Slide 1
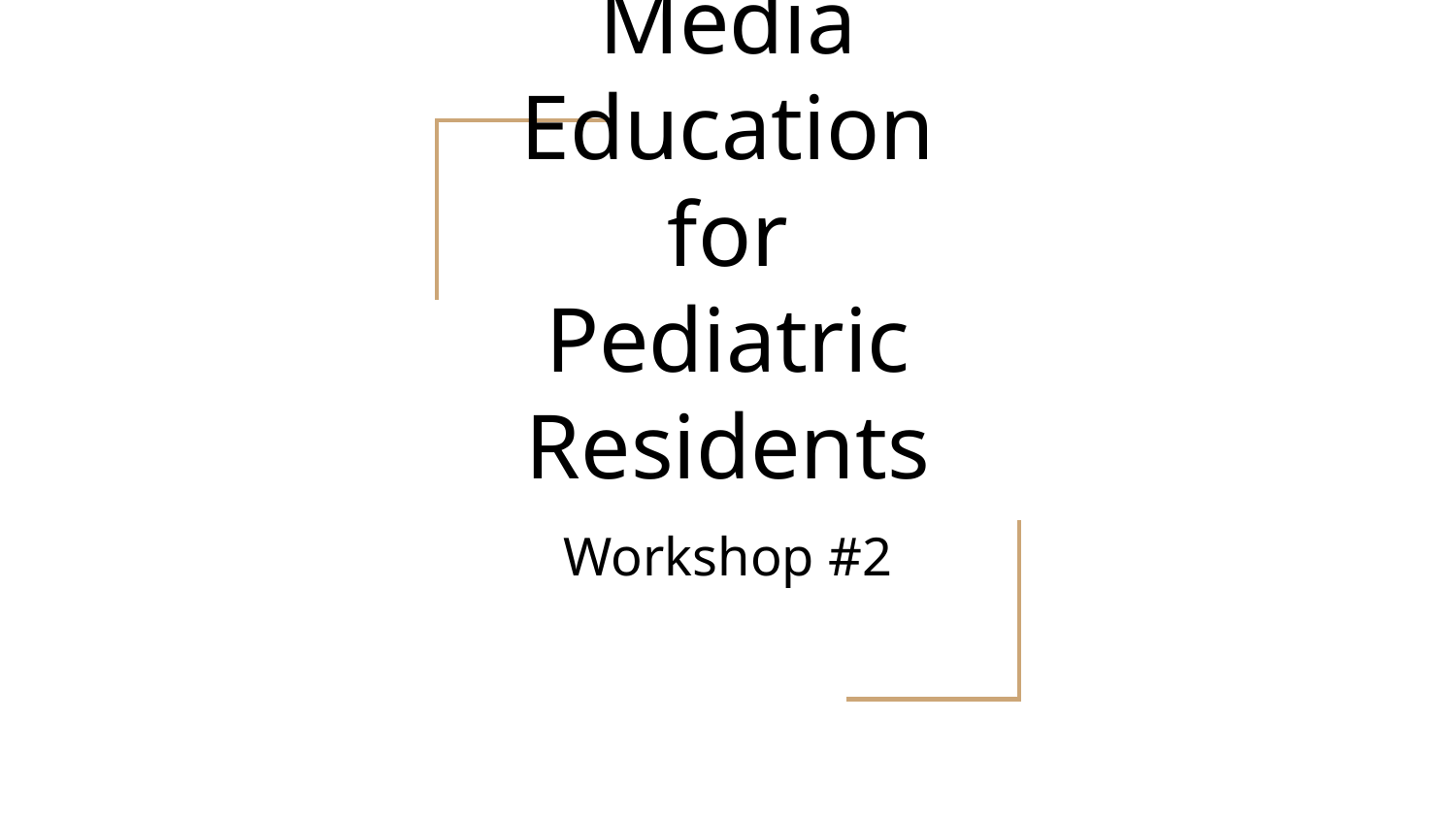

# Media Education for Pediatric Residents
Workshop #2

## Slide 2
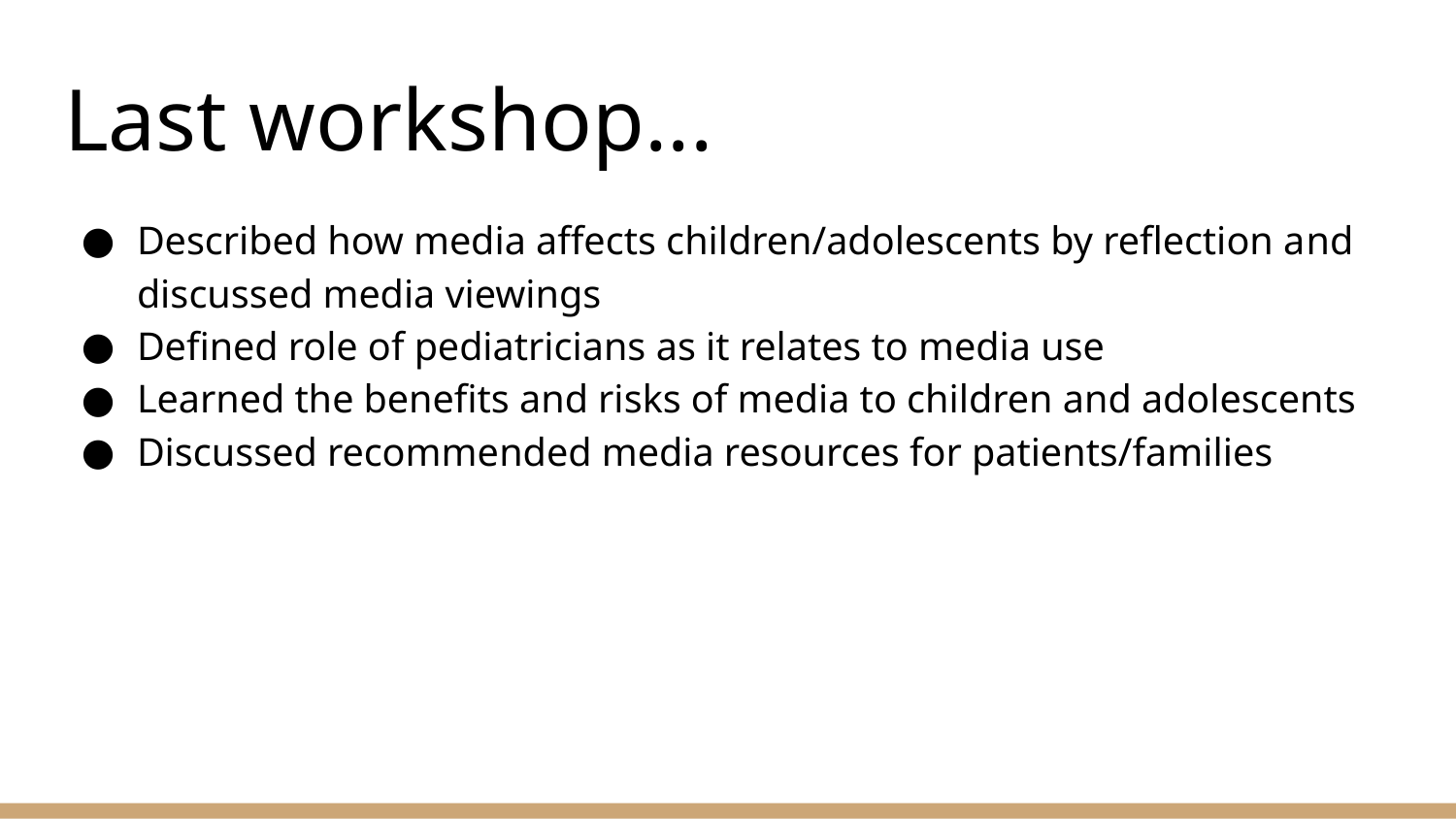

# Last workshop...
Described how media affects children/adolescents by reflection and discussed media viewings
Defined role of pediatricians as it relates to media use
Learned the benefits and risks of media to children and adolescents
Discussed recommended media resources for patients/families

## Slide 3
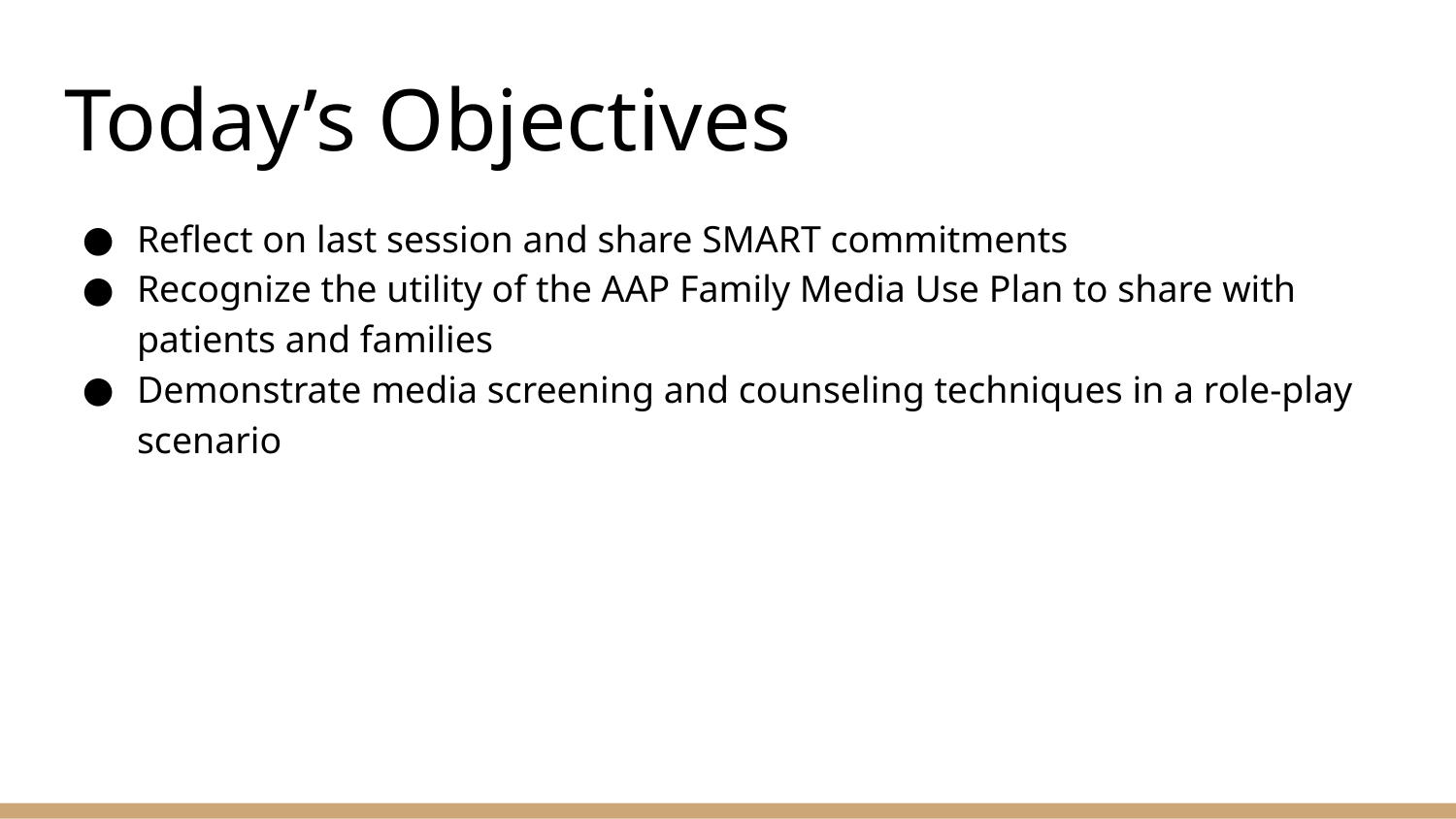

# Today’s Objectives
Reflect on last session and share SMART commitments
Recognize the utility of the AAP Family Media Use Plan to share with patients and families
Demonstrate media screening and counseling techniques in a role-play scenario

## Slide 4
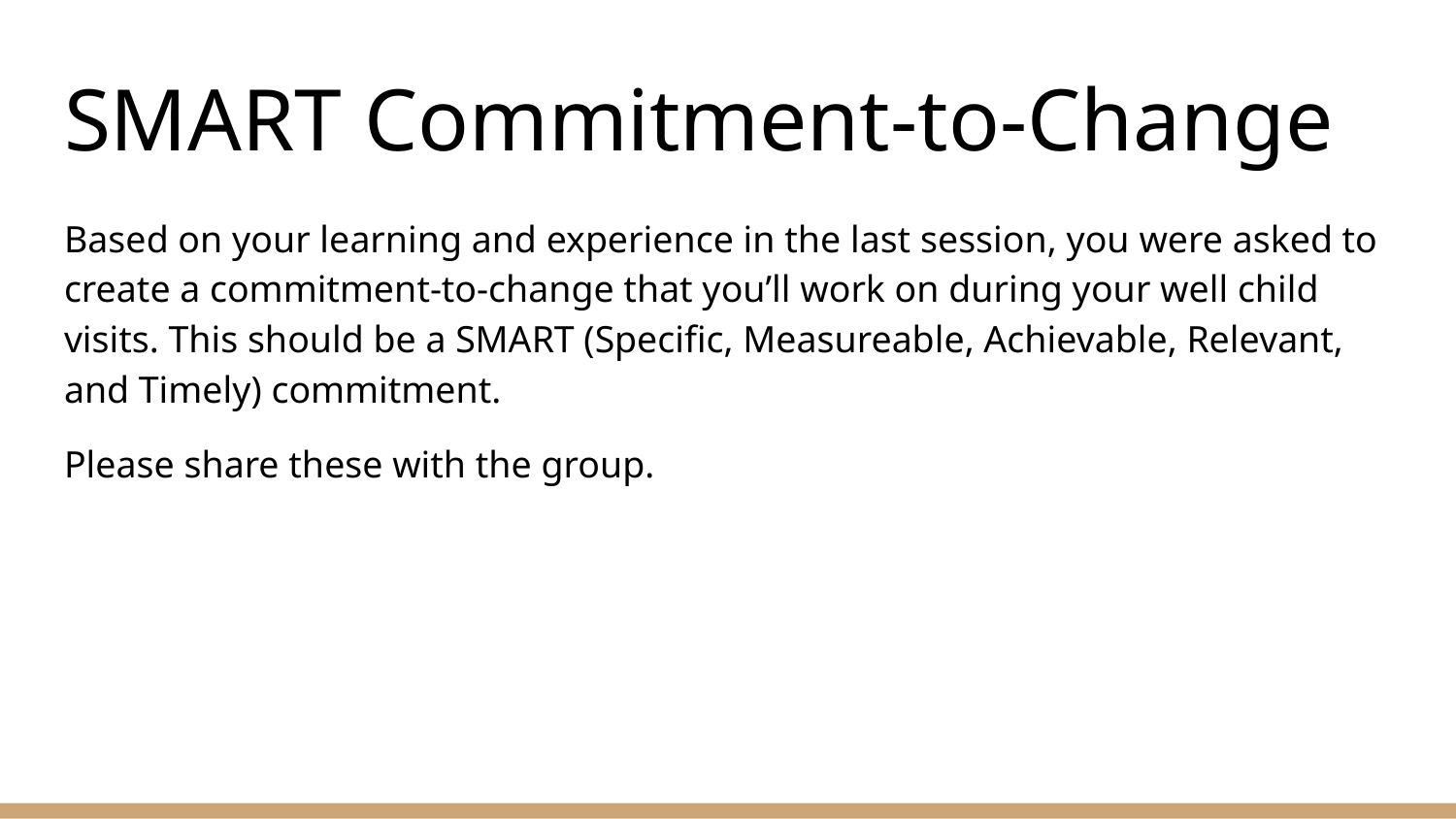

# SMART Commitment-to-Change
Based on your learning and experience in the last session, you were asked to create a commitment-to-change that you’ll work on during your well child visits. This should be a SMART (Specific, Measureable, Achievable, Relevant, and Timely) commitment.
Please share these with the group.

## Slide 5
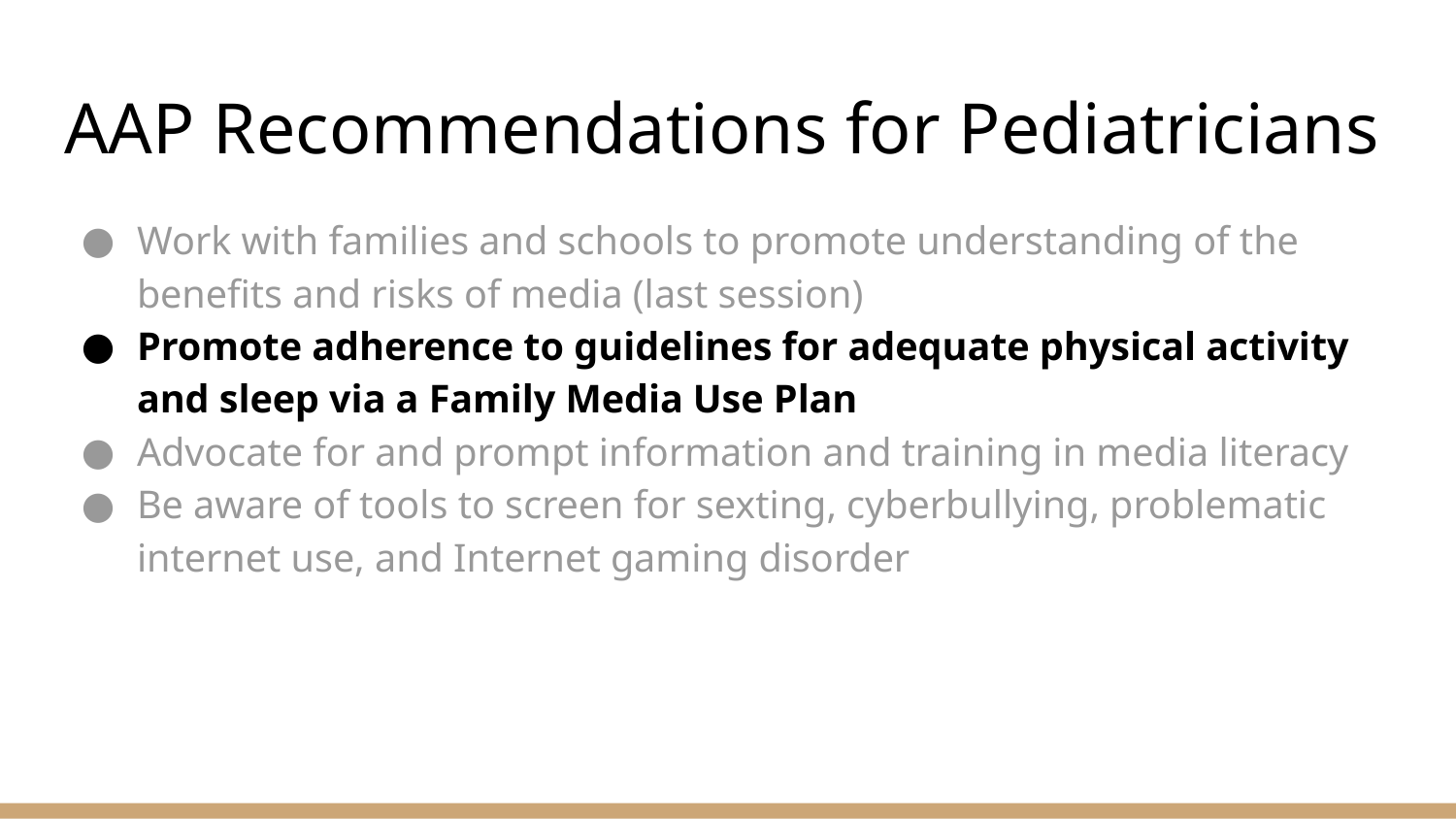

# AAP Recommendations for Pediatricians
Work with families and schools to promote understanding of the benefits and risks of media (last session)
Promote adherence to guidelines for adequate physical activity and sleep via a Family Media Use Plan
Advocate for and prompt information and training in media literacy
Be aware of tools to screen for sexting, cyberbullying, problematic internet use, and Internet gaming disorder

## Slide 6
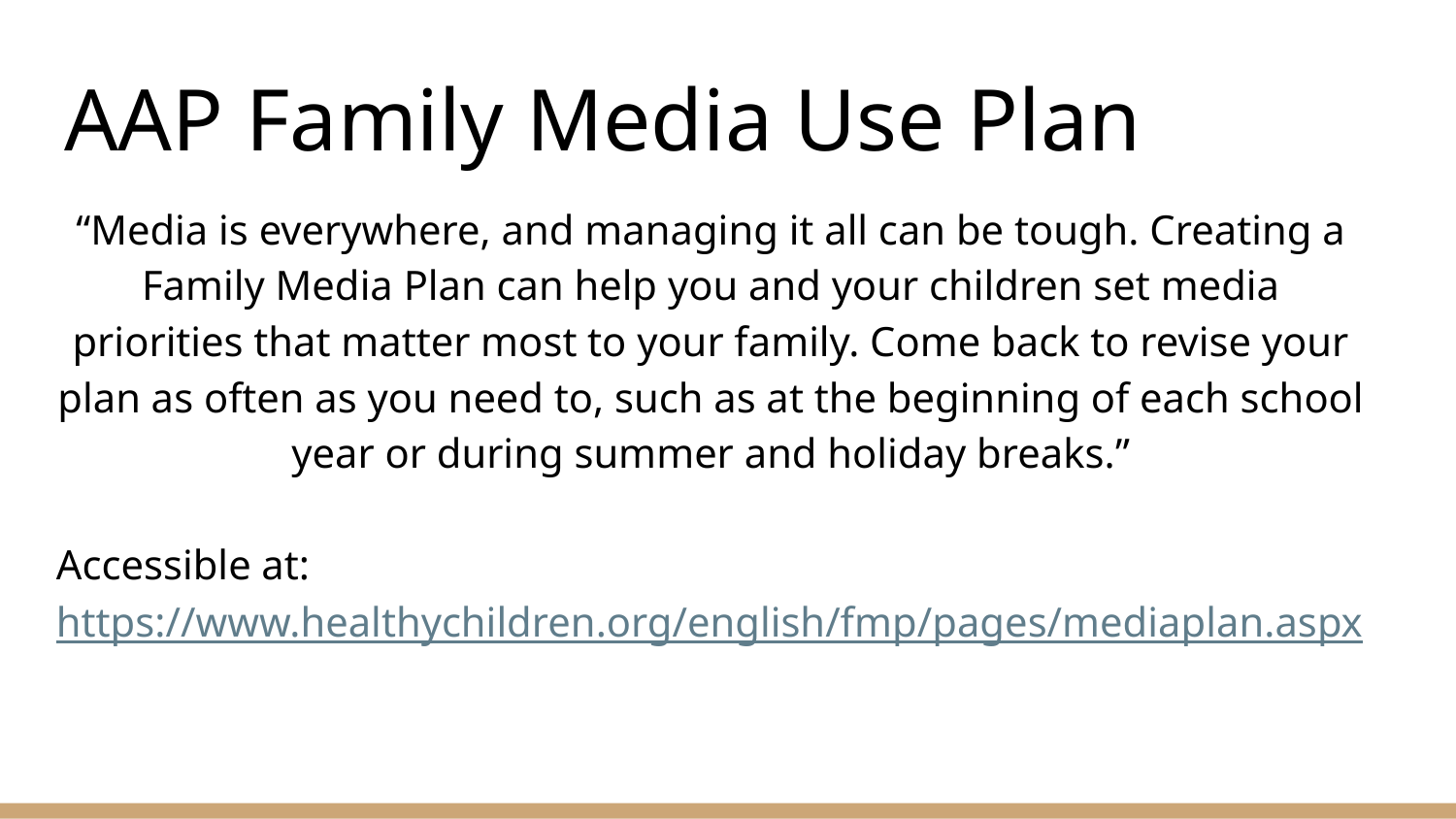

# AAP Family Media Use Plan
“Media is everywhere, and managing it all can be tough. Creating a Family Media Plan can help you and your children set media priorities that matter most to your family. Come back to revise your plan as often as you need to, such as at the beginning of each school year or during summer and holiday breaks.​”
Accessible at: https://www.healthychildren.org/english/fmp/pages/mediaplan.aspx

## Slide 7
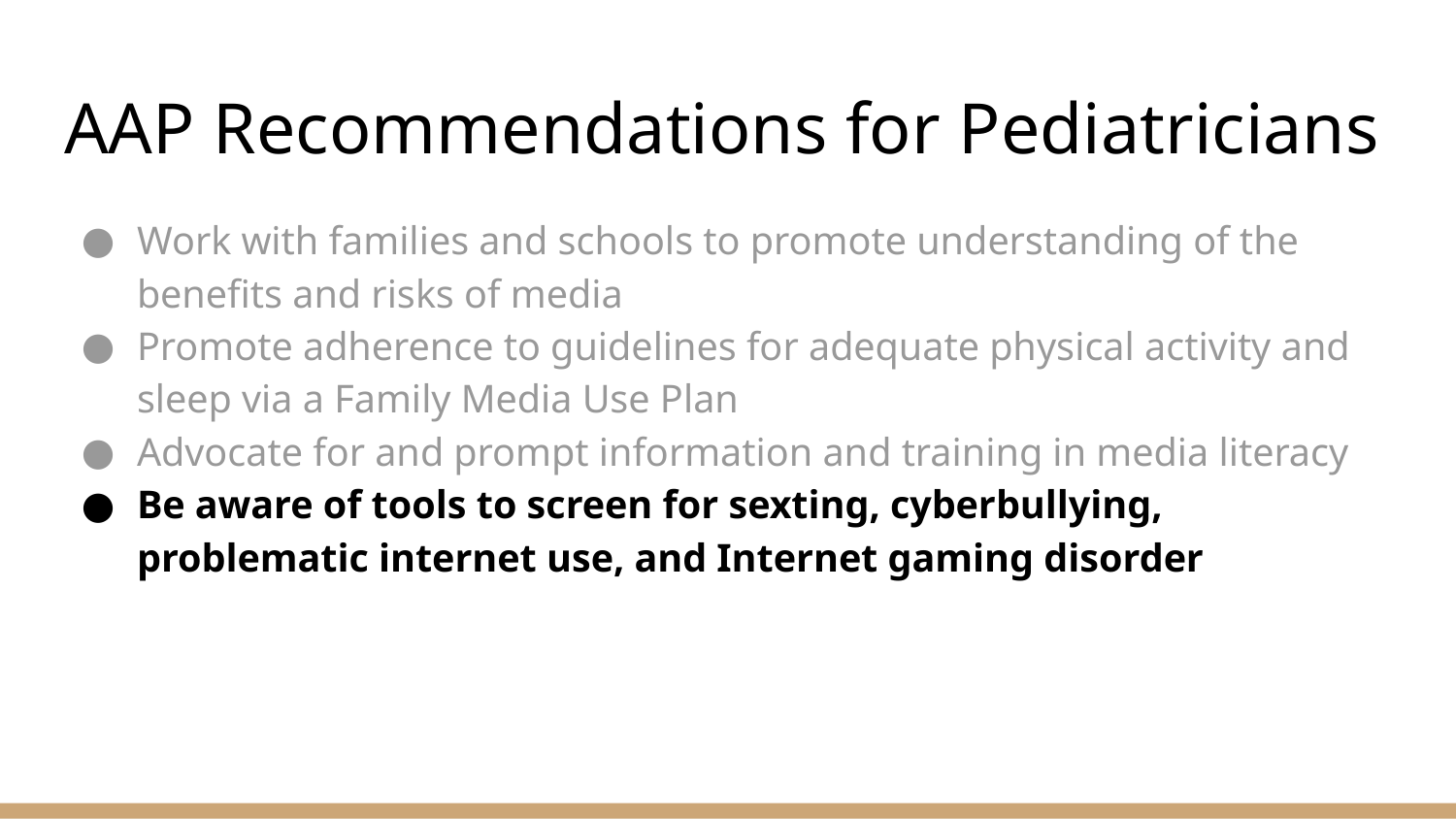

# AAP Recommendations for Pediatricians
Work with families and schools to promote understanding of the benefits and risks of media
Promote adherence to guidelines for adequate physical activity and sleep via a Family Media Use Plan
Advocate for and prompt information and training in media literacy
Be aware of tools to screen for sexting, cyberbullying, problematic internet use, and Internet gaming disorder

## Slide 8
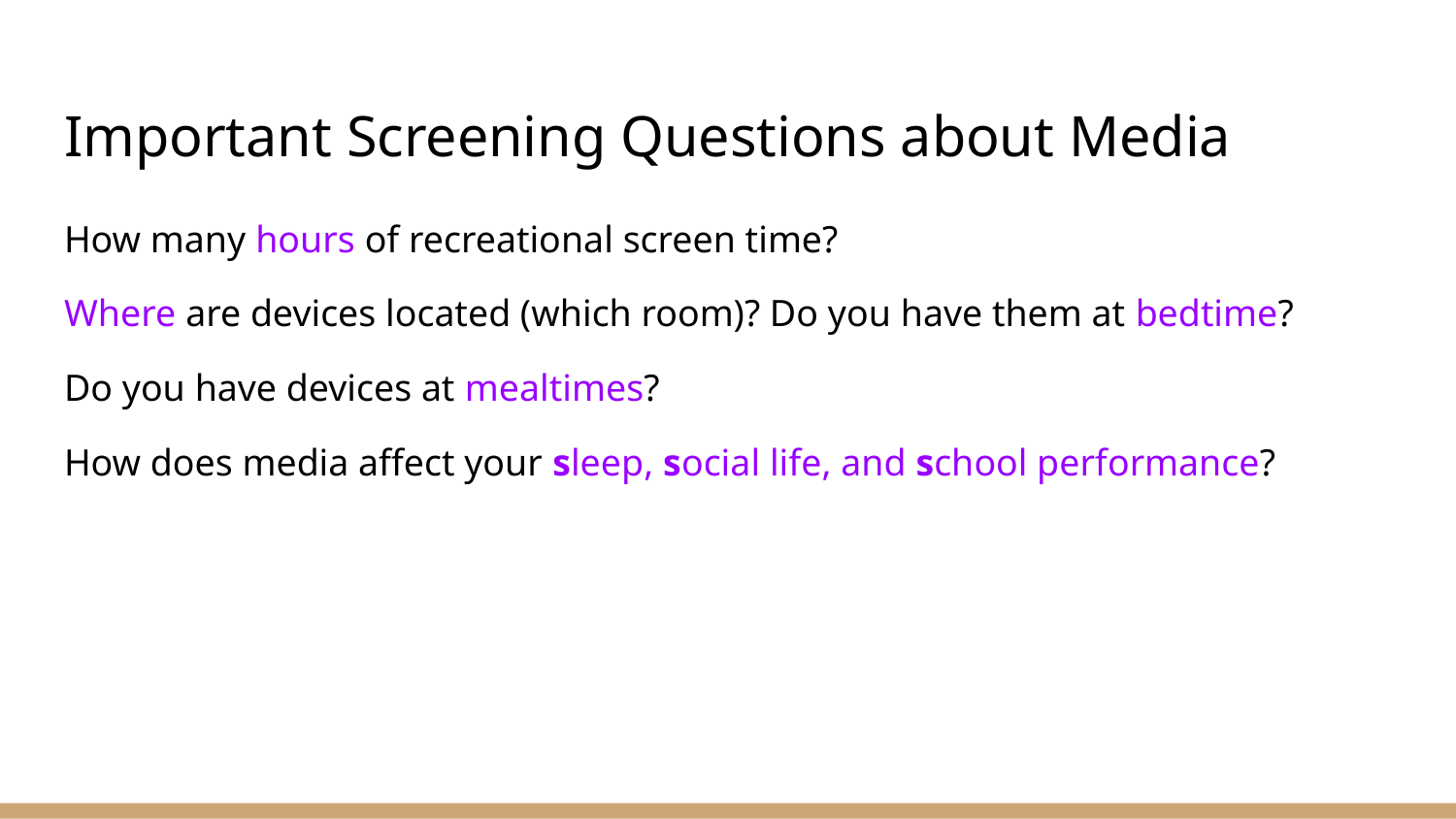

# Important Screening Questions about Media
How many hours of recreational screen time?
Where are devices located (which room)? Do you have them at bedtime?
Do you have devices at mealtimes?
How does media affect your sleep, social life, and school performance?

## Slide 9
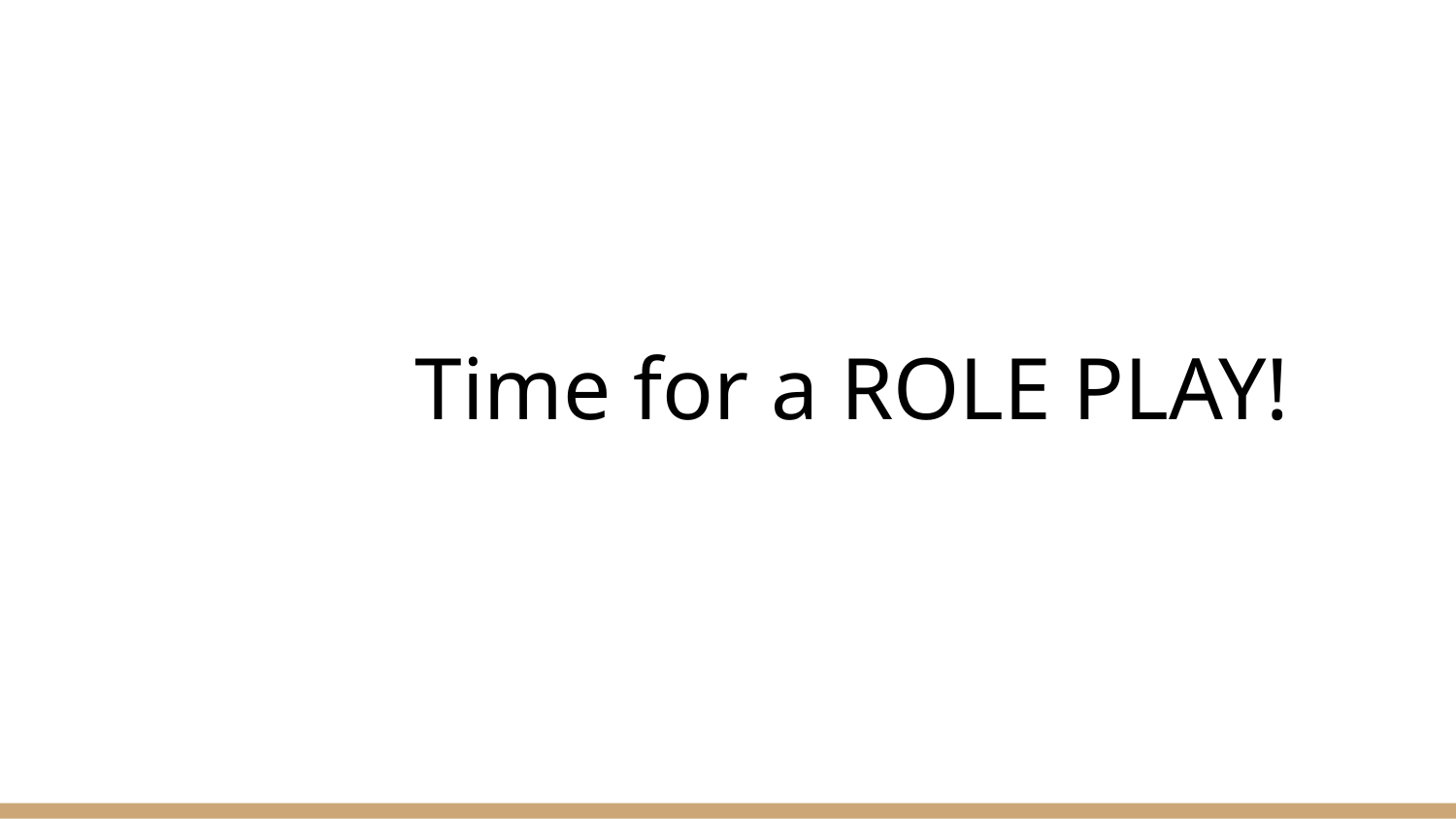

# Time for a ROLE PLAY!

## Slide 10
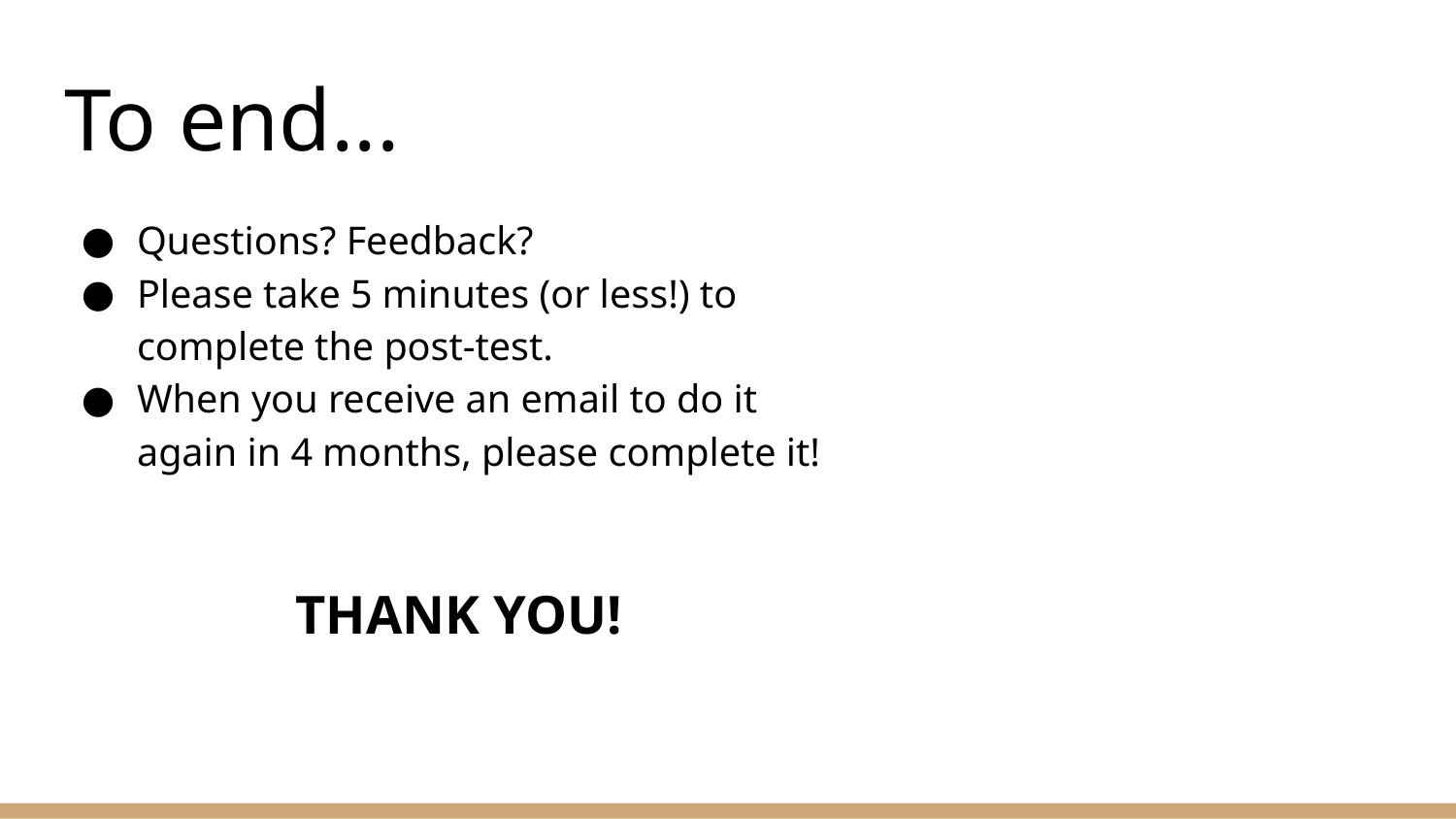

# To end...
Questions? Feedback?
Please take 5 minutes (or less!) to complete the post-test.
When you receive an email to do it again in 4 months, please complete it!
THANK YOU!
