# Supplementary material for: Implementation and Evaluation of a Novel Media Education Curriculum for Pediatric Residents
Source: MedEdPORTAL. 2023 Dec 22;19:11372. doi: 10.15766/mep_2374-8265.11372 (PMC10739037; doi:10.15766/mep_2374-8265.11372)
Supplement: Supplementary file 1 — Timeline for Curriculum.docxPretest.docxWorkshop 1 Slides.pptxWorkshop 2 Slides.pptxRole-Play Patient Script.docxRole-Play Physician Guide.docxRole-Play Observation of Performance Checklist.docxPosttest Immediately After Curriculum.docxPosttest 4 Months After Curriculum.docxAnswer Key to Knowledge Questions.docx [file mep_2374-8265.11372-s001.zip › A. Timeline for Curriculum.docx]

| Time  **Appendix A: Timeline for Curriculum** | Activity | Comments | Location |
| --- | --- | --- | --- |
| **Prior to 1^st^ Session** |  |  |  |
| 0-2 weeks before | Assignment emailed: Media Viewing of Participant’s’ choice that includes adolescent topics | Recommended Viewings:  1. *The Social Dilemma* - Documentary about social media’s integration and consequences on society, particularly youth  2. *Love, Simon* – Coming-of-age movie including themes of LGBTQ+ youth, bullying, social media, sexuality, and inclusivity  3. *Perks of Being a Wallflower* – Coming-of-age moving including themes of independence, sexuality, substance use, trauma, eating disorders  4. *The Mask You Live In –* Documentary about how boys are directed to become “men,” including themes of sexuality, abuse, suicide, sexism, substance use, role models, empathy  *Highly recommend that facilitator watch all viewings to best guide discussion but all are not required to meet learning objectives* For participants, these are recommended (not mandatory) as ability to freely view will likely vary among participants* | Independent |
| 0-2 weeks before | While watching, write down thoughts to reflect independently. | Questions to guide reflection:  -How did watching this make you feel?  -How would an adolescent feel differently if watching this?  -Do you think this viewing correctly reflects adolescent experiences with media? Why or why not?  Optional recommended resource for participants to type independent reflection:  www.padlet.com | Independent |
| **1^st^ session** |  |  |  |
| 5 minutes | Introduction of curriculum objective and session learning objectives | Led by instructor using two slides | Zoom meeting room utilizing screenshare feature |
| 20 minutes | Small group discussion using independent reflections | Facilitator helps guide discussion using prompts if needed:  -What are potential benefits and risks to media use?  -How can this movie be used as a tool to educate patients/families?  -What interventions did you learn from the viewing that you could counsel patients on regarding media use? | Zoom meeting room utilizing screenshare feature |
| 5 minutes | Introduction of American Academy of Pediatrics (AAP) Policy Statement | Instructor to introduce the AAP policy statement “Media Use in School-Aged Children and Adolescents” with focus on AAP recommendations for pediatricians | Zoom meeting room utilizing screenshare feature |
| 5 minutes | WordCloud activity using Poll Everywhere available on Google Slides | Participants respond to prompt “What are the benefits and risks of media to child and adolescent health (Use 1-2 word answers without spaces)?” and responses show up in word cloud  Option: Use Zoom chat feature if Poll Everywhere Word Cloud not an option | Zoom meeting room utilizing screenshare feature |
| 20 minutes | Review of Media Literature | -Facilitator to review benefits and negative effects of media use to adolescents as outlined by AAP, and AAP recommendations for families  -Facilitator to introduce recommended resources to use for counseling including:  1. AAP Family Media Plan - https://www.healthychildren.org/english/fmp/pages/mediaplan.aspx  2. ©Common Sense Media - https://www.commonsensemedia.org/  3. ©Digital Wellness Lab - https://digitalwellnesslab.org/ | Zoom meeting room |
| 5  minutes | Questions, feedback, assignment | Allow time to learners to ask questions, reflect on session, provide feedback. Participants assigned to create a SMART Commitment to Change to work on personally or professionally to bring to next session, based on this session’s topics. | Zoom meeting room |
| **2^nd^ session** |  |  |  |
| 5 minutes | Debrief of 1st session | Allow time to learners to ask questions, reflect on previous session. Introduce today’s objectives. | Zoom meeting room |
| 5 minutes | Share Commitment to Change | Participants share their assignments with group. | Zoom meeting room |
| 20 minutes | Review of AAP Recommendations for Pediatricians now with focus on screening and counseling | Facilitator to review sample AAP Family Media Plan to teach important screening questions and counseling strategies.  Facilitator to review other recommended resources available on https://www.commonsensemedia.org/ and https://digitalwellnesslab.org/ | Zoom meeting room utilizing Screenshare feature |
| 20 minutes | Role-play scenario | Participants partake in role play scenario. One participant is the “pediatrician” actor, one participant is the “patient” actor. Script will be provided to the “patient.” The “pediatrician” will complete a HEEADSSS assessment and incorporate screening and counseling about media use.  -Instructor (and if 3^rd^ participant present) will observe role play scenario and fill out Observation of Performance Checklist to assess performance | Zoom meeting room |
| 10 minutes | Debrief of role-play scenarios | Allow learners to self-assess their performance and ask questions. Instructor to moderate discussion. Review Observation of Performance Checklist. | Zoom meeting room |
| 10 minutes | Questions, feedback | Allow time to learners to ask questions, reflect on session, provide feedback. | Zoom meeting room |
