# Supplementary material for: Implementation and Evaluation of a Novel Media Education Curriculum for Pediatric Residents
Source: MedEdPORTAL. 2023 Dec 22;19:11372. doi: 10.15766/mep_2374-8265.11372 (PMC10739037; doi:10.15766/mep_2374-8265.11372)
Supplement: Supplementary file 1 — Timeline for Curriculum.docxPretest.docxWorkshop 1 Slides.pptxWorkshop 2 Slides.pptxRole-Play Patient Script.docxRole-Play Physician Guide.docxRole-Play Observation of Performance Checklist.docxPosttest Immediately After Curriculum.docxPosttest 4 Months After Curriculum.docxAnswer Key to Knowledge Questions.docx [file mep_2374-8265.11372-s001.zip › _Educational Summary Report_11372.pdf]

MedEdPORTAL® The AAMC Journal of Teaching and Learning Resources

Original Publication

Open Access

Implementation and Evaluation of a Novel Media Education Curriculum for Pediatric Residents

Rashi Kabra, MD, MSEd*, Shara Steiner, DO, MACM, Jane Cerise, PhD, Nadia Saldanha, MD

*Corresponding author: kabrar@chop.edu

Abstract

Introduction: Despite increasing awareness of media exposure to children and adolescents and the known value of media education for physicians, residency programs lack formal media education. **Methods:** We designed an interactive curriculum for pediatric residents to teach health effects of media as well as screening and counseling strategies. Instructional methods were based on constructivism, experiential learning, and situated learning theories. Participants independently reflected on a media viewing, then participated in two facilitator-led 1-hour workshops of two to three residents. Facilitators received speaker notes based on American Academy of Pediatrics media guidelines. Changes in knowledge, reported skills, and attitudes were assessed by pre- and posttests. **Results:** Twenty-one residents completed the curriculum from September 2021 through April 2022. Knowledge improved after the curriculum as the median score increased from 3 to 5 out of 6, although 4 months later it was insignificant. Reported skills in screening did not significantly change. Residents strongly agreed that media use was an important health issue, with medians of 9 or 10 out of 10 on all tests. Attitudes regarding residency preparedness and confidence in screening and counseling significantly improved from pretest medians of 6 and 6 out of 10, respectively, to posttest medians of 8 and 9 to 4-month posttest medians of 6 and 8. **Discussion:** A media curriculum for pediatric residents resulted in improved knowledge and attitudes. Enhanced attitudes demonstrated sustainability. All participants found the curriculum relevant and engaging and felt it should be continued.

Keywords
Anticipatory Guidance, Social Media, Adolescent Medicine, Curriculum Development, Online/Distance Learning, Pediatrics

Educational Objectives

By the end of this activity, learners will be able to:

1. Describe media-related health benefits and risks in children and adolescents as outlined by the American Academy of Pediatrics.
2. Demonstrate media screening and counseling techniques using role-play that apply to children and families presenting to a pediatrician.
3. Provide and discuss with patients and families access to web-based resources they can use at home regarding healthy and unhealthy media practices.

Citation:
Kabra R, Steiner S, Cerise J, Saldanha N. Implementation and evaluation of a novel media education curriculum for pediatric residents. *MedEdPORTAL.* 2023;19:11372.
https://doi.org/10.15766/mep_2374-8265.11372

Introduction

The American Academy of Pediatrics (AAP) recognizes that media presents both health benefits and risks to children and adolescents.[1] Benefits of media include contributions to education and creativity, increased awareness of current events, increased civic engagement, enhanced support networks, and social inclusion.[1,2] Negative effects of media among adolescents include obesity, aggressive behavior and trauma secondary to media violence, risky sexual behavior, normalization of substance use, body image distortion, decreased school achievement, sleep disturbances, and decreased propensity for interpersonal relationships.[1,2]

Despite increasing awareness of media exposure and the known value of media education,[2] residency programs lack formal education about media effects on child and adolescent health. One study found that although 91% of graduating pediatric residents believed media use was a health issue for children and adolescents, only 38% rated their program adequate in preparing them to provide anticipatory guidance about media exposure and media effects.[3] Another study found that less than

Copyright © 2023 Kabra et al. This is an open-access publication distributed under the terms of the Creative Commons Attribution license.

one-third of U.S. pediatric residency programs teach about media exposure.[4]

The current adolescent medicine curriculum nationwide involves understanding and practice of the HEEADSSS (home, education/employment, eating, activities, drugs, sex, suicidality, and safety) assessment to screen for risky behaviors and protective factors.[5] The assessment may include a question about hours of recreational screen time but lacks additional questions about media usage. Current literature supports education of physicians and medical trainees about personal media use and its professional relevance yet lacks any educational tools to teach patients about media use.[6-8] Pediatric residents are therefore not formally taught to explore specific exposures to media and how these exposures can affect adolescent health or to teach healthy and unhealthy media practices to patients and families.

This curriculum addresses that educational gap by teaching pediatric residents the prevalent health and behavioral effects of media, how to screen for media exposure, and how to counsel. Learners are encouraged to screen and counsel patients about media during the activities portion of the HEEADSSS assessment. The curriculum has been designed based on constructivism and the experiential theory of learning, as learners are asked to reflect on their own media experiences to construct meaning and integrate new information with previous knowledge. The small-group sessions within the curriculum also rely on the situated learning theory, as the knowledge that learners gain is largely dependent on their interactive discussion and activities. Learners use the small group as a method to think aloud, share their reflections, ask each other questions, and learn together through the experience.[9] The curriculum is an ideal strategy for health professions educators because it is a novel method to teach media education to pediatric residents who are treating a generation growing up fully immersed in media.

Methods

This curriculum was targeted at a convenience sample of pediatric residents from a large residency program in the New York metropolitan area who rotated through adolescent medicine from September 2021 through April 2022. Residents were excluded if they did not participate in both small-group workshops due to the importance of continuity from the first through the second workshop. This educational innovation was approved by the Northwell Health Institutional Review Board.

A timeline for the curriculum can be found in Appendix A. Prior to the first workshop, we sent participants an email containing an introduction to the curriculum and instructions to complete a prework assignment and pretest. The prework was one viewing from a list of movies or documentaries that were chosen based on inclusion of adolescent themes including social media use, mental health, sexuality, gender identity, substance use, eating disorders, trauma, and independence. During the viewing, participants were asked to reflect independently, utilizing guiding prompts found in Appendix A. The pretest assessed preliminary knowledge of health benefits and risks of media use, current skills and attitudes about media screening and counseling, and perceptions of media training provided by residency (Appendix B). We created the pretest based on media recommendations from the AAP, as there were no current validated instruments to assess resident knowledge, skills, and attitudes about this topic.

The first small-group session took place 1-2 weeks into the rotation on the Zoom virtual conference platform. We asked all participants to have their cameras and microphones turned on. The 60-minute session included the facilitator and two to three pediatric residents. The facilitator needed to have prerequisite knowledge of the current AAP policy statement on media use in school-age children and adolescents[2] and familiarity with recommended media resources as denoted in the speaker notes of the associated slide deck (Appendix C). For the first 5 minutes, the instructor introduced the curriculum and session learning objectives. For the next 20 minutes, participants debriefed their media viewings and shared personal reflections.

The next component of the first session was a review of important media literature, with a focus on the AAP policy statement on media.[2] After introduction of the policy statement and review of the AAP's recommendations for pediatricians, through a word cloud activity we asked participants, "What are the benefits and risks of media to child and adolescent health?" The instructor reviewed responses, then used the lists on the next two slides to discuss any benefits or risks that had not already been mentioned. The instructor then introduced several recommended resources to use with patients, including the AAP Family Media Plan,[10] CommonSenseMedia.org,[11] and DigitalWellnessLab.org.[12] The last 5 minutes of the session were reserved for learners to ask questions, reflect on the session, and provide feedback. We then assigned learners to create a SMART (specific, measurable, achievable, relevant, and timely) commitment to change based on the session's topics and instructed them to bring it to the next session.

The second 60-minute session (Appendix D) started with a 5-minute debrief of the first session, followed by an overview of the current session's objectives. The next 5 minutes were spent with participants sharing their SMART commitments. A

Copyright © 2023 Kabra et al. This is an open-access publication distributed under the terms of the Creative Commons Attribution license.

20-minute didactic session followed where the instructor reviewed screening and counseling strategies by introducing the AAP Family Media Plan[10] and encouraging use of other recommended resources with media handouts for patients and clinicians.[12] Next, participants did a role-play scenario in which the participant acting as a physician was tasked with completing a HEADSSS assessment on the patient incorporating media screening questions and counseling strategies. We provided the participant acting as the patient with a script (Appendix E) and the participant acting as the physician with a guide (Appendix F). If another participant was present, they were assigned to be an observer and given an Observation of Performance checklist to rate the scenario along with the facilitator (Appendix G). Though the role-play activity was not used for data collection, the scenario was followed by a debrief in which learners self-assessed their performance and the observer and/or facilitator shared the Observation of Performance checklist with the participants.

During the final 10 minutes, learners received an electronic posttest to complete (Appendix H). This posttest evaluated knowledge gained on benefits and risks of media use to children and adolescents, self-reported skills in asking specific media screening questions, perception of media training provided by residency thus far, perception about media use and counseling, and satisfaction with the curriculum. Four months after curriculum completion, we disseminated another posttest, which again assessed knowledge, skills, and attitudes (Appendix I). The answer key to the knowledge-based questions on the pre- and posttests (the same questions and answers on all three tests) can be found in Appendix J.

Data were collected from completed pre- and posttests and entered into SAS for statistical analysis. Descriptive statistics with frequencies and percentages were used to summarize categorical variables. Medians and interquartile ranges (IQRs) were used to summarize continuous variables. Changes in knowledge and reported skills and attitudes of pediatric residents were assessed by pre- and posttests immediately after the curriculum and 4 months later using Kruskal-Wallis tests followed by Wilcoxon rank sum tests for continuous variables and Fisher's exact test for categorical variables. Analyses were performed treating pre- and posttests as independent, as the anonymous nature of the survey prevented the pairing of tests completed by the same individual. A p value of .05 was considered significant. For post hoc paired comparisons between pre- and posttest and pre- and 4-month posttest, a p value of .025 was considered significant after Bonferroni adjustment. For each survey period, Spearman correlation coefficients were calculated between all pairs of knowledge, skills, attitudes about importance, attitudes about residency preparedness, and attitudes about confidence scores.

Results

Twenty-one pediatric residents completed the media education curriculum from September 2021 through April 2022. All participants completed a pre- and immediate posttest (Appendices B and H), and 15 participants completed the 4-month posttest (Appendix I). Participant demographics can be found in Table 1.

Residents were asked six knowledge-based multiple-choice questions addressing benefits, risks, and current media recommendations in pediatrics, as well as three 5-point Likert-scale questions (1 = *never,* 5 = *always*) assessing current skills when screening patients about media. There were also six 5-point Likert-scale questions (1 = *strongly disagree,* 5 = *strongly agree*) assessing attitudes divided into three themes, including media importance, residency preparedness, and confidence with screening and counseling patients. The posttests contained three additional questions asking whether the curriculum was relevant, was engaging, and should be continued. The 4-month posttest included additional screening and counseling questions that had been specifically taught during the curriculum. Descriptive statistics including medians and IQRs can be found in the Figure and Table 2.

A total knowledge score was calculated based on the number of correct answers out of six. Median knowledge score significantly

Table 1. Participant Demographics

Demographic	Participants Completing Pretest and Posttest (N = 21) No.	%	Participants Completing 4-Month Posttest (N = 15) No.	%
Academic year				
PGY 2	16	76	13	87
PGY 3	5	24	2	13
Gender				
Female	12	57	9	60
Male	9	43	6	40
Career plans				
General pediatrics practice	3	14	3	20
Pediatric subspecialty fellowship	16	76	10	67
Chief residency	1	5	1	7
Other position	0	0	0	0
Undecided	1	5	1	7
Prior media education				
Yes	5	24	2	13
No	14	67	11	73
I don't know	2	10	2	13

Copyright © 2023 Kabra et al. This is an open-access publication distributed under the terms of the Creative Commons Attribution license.

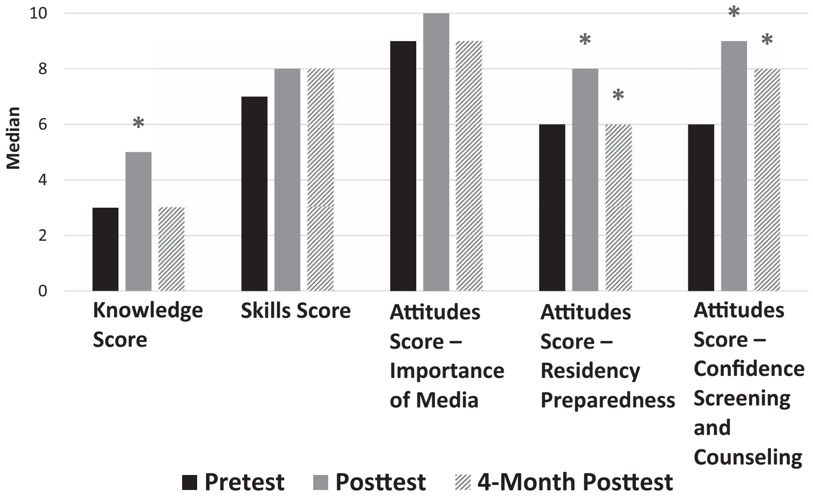

Figure. Median scores on pretest, posttest, and 4-month posttest. Asterisk (*) indicates $p < .025$ when compared to pretest. Knowledge score was based on the number of correct answers out of six multiple-choice questions. Skills score was the sum of the scores on three questions, each rated on a 5-point Likert scale (1 = *never,* 5 = *always*), with a maximum possible score of 15. Attitudes scores were the sum of the scores on two questions, each rated on a 5-point Likert scale (1 = *strongly disagree,* 5 = *strongly agree*), with a maximum possible score of 10.

improved from 3 (IQR, 2-3) on the pretest to 5 (IQR, 4-5) on the posttest. Four months later, median knowledge score decreased to 3 (IQR, 3-5; $p = .11$), which was not a significant change when compared to the pretest.

Likert-scale questions were grouped into similar themes, and total scores were calculated as continuous variables based on 5-point responses. When assessing skills, three Likert-scale questions were grouped together for a composite possible score of 15. Residents scored low on the pretest, with a median score of 7 (IQR, 6-9), and improved to a median score of 8 (IQR, 7-9) on the posttest and 8 on the 4-month posttest (IQR, 6-11), though both posttest scores were not significant when compared to the pretest score.

When evaluating specific attitudes, we grouped two Likert-scale questions together for a composite possible score of 10 for each attitude theme. Before and after the curriculum, residents strongly agreed that media was important and media counseling by pediatricians was important and necessary, with median scores of either 9 or 10 out of 10 on all three tests. While attitudes regarding residency preparedness and confidence screening and counseling prior to the curriculum were low, with medians of 6 (IQR, 4-6) and 6 (IQR, 6-7) out of 10, respectively, significant

Table 2. Reactions to Curriculum and Correlations

Descriptive Statistic	*Mdn*	Lower Quartile	Upper Quartile	Pearson's *r*	*p*[a]
Reactions to curriculum[b]					
I feel that this media education curriculum is relevant to my job.					
Posttest	5	4	5		
4-month posttest	5	4	5		
I feel that this media education curriculum was engaging.					
Posttest	5	4	5		
4-month posttest	5	4	5		
I feel that this media education curriculum should be provided to all pediatric residents.					
Posttest	5	4	5		
4-month posttest	5	4	5		
Correlation between attitudes scores on residency preparedness and confidence screening and counseling					
Pretest				.76	<.001
Posttest				.43	.05
4-month posttest				.43	.11

[a]Significant at $p < .025$.
[b]Rated on a 5-point Likert scale (1 = *strongly disagree,* 5 = *strongly agree*).

Copyright © 2023 Kabra et al. This is an open-access publication distributed under the terms of the Creative Commons Attribution license.

changes were found immediately after the curriculum, with median improving to 8 (IQR, 7-8) on residency preparedness and 9 (IQR, 8-9) on confidence with screening and counseling. Four months later, attitudes about residency preparedness and confidence screening and counseling remained significantly improved, with medians of 6 (IQR, 6-8) and 8 (IQR, 8-8; $p =$ .002), respectively. Questions unique to the posttest and 4-month posttest assessing curriculum relevance, engagement, and continuation on a 5-point scale demonstrated the maximum score, with medians of 5 (IQR, 4-5) on all three questions on both tests.

Correlation studies were done to assess whether relationships existed between knowledge, skills, and attitudes with regard to media. There was a significant positive correlation found between residency preparedness and confidence screening and counseling on the pretest ($r = .764$, $p < .01$), while the posttest correlation was $r = .431$ ($p = .05$). Other correlation studies were not found to be significant.

Feedback on the curriculum during and after the sessions was widely positive. One resident commented, “I found this helpful, short, succinct, and not dragged out with a million modules. These days less is more. Because it was two brief sessions, I was able to pay attention.” Another resident stated, “This was very informational. Will try to incorporate some of the techniques that we learned when asking and counseling patients.” Some residents requested additional training: “This was an excellent curriculum! I would love even more training, including more about the risks and benefits to adolescents.”

Discussion

This curriculum addressed an important educational gap in pediatric residency training about media use as a health issue. Prior to the curriculum, residents demonstrated inadequate knowledge about health effects of media and reported suboptimal screening skills. Residents strongly agreed that media use was a health issue in pediatrics and that media counseling by pediatricians was necessary and important, yet they reported poor attitudes about residency training in this topic and inadequate perceived confidence with screening and counseling.

Immediately after the curriculum, residents demonstrated a significant increase in knowledge about media’s health effects, though this change was not sustained 4 months later. There was no significant change noted in reported skills in media screening. Data also revealed significant increases in attitudes about residency preparedness on this topic and confidence screening and counseling that remained significant 4 months later. Lastly, all participants found the media curriculum relevant and engaging and felt it should continue to be offered. This curriculum therefore addresses Kirkpatrick evaluation levels of reaction and learning,[13] though learning was not sustained.

Throughout the curriculum, residents commented on the importance of this content and its current relevance to their patient population. They highlighted the need for continued media education, which could lead to longitudinal learning. The facilitator found that incorporation of several active learning strategies interspersed with minimal passive learning kept learners engaged and promoted dynamic discussion. Though the curriculum was implemented virtually, all curricular aspects can be done in person, which may facilitate more robust discussion. Another lesson learned is the need for facilitators to familiarize themselves with the continually growing literature in this field. Finally, participants indicated that direct observation with patients could be useful in skill building, and the investigators agree that Kirkpatrick level 3 could be addressed by direct observation during patient encounters.[13]

This educational innovation does have some limitations. Single-site implementation and small sample size limited generalizability of findings. The survey was created by the investigators and was not a validated tool as one does not currently exist in literature. This limitation raises the question of survey validity, and the sample size was not large enough to run statistical analysis to support internal reliability within survey items. Though participants were informed of anonymity, there may have been a response bias in the form of a social desirability bias when reporting skills, leading to overestimation. Inclusion of Likert-scale questions may have also introduced neutral response bias. Lastly, although each record in the pretest data had a corresponding record in the posttest data, identifying information was not collected that would enable paired analysis. Similarly, because the 4-month posttest dataset had responses from 71% (15 of 21) of the residents, it is unclear how respondents in the 4-month group were different from the full cohort. Therefore, we used the Wilcoxon rank sum test under the assumption that pre- and posttest datasets were independent and that it would be the optimal test given the available data.

Despite these limitations, this curriculum has important implications for residency education. We found that pediatric resident knowledge and attitudes regarding media use improved with implementation of an interactive virtual media curriculum. Incorporation of active learning strategies encouraged participation and interaction between peers. Residents frequently

Copyright © 2023 Kabra et al. This is an open-access publication distributed under the terms of the Creative Commons Attribution license.

contributed to discussion by sharing and reflecting on their own media experiences. Future research can target methods to ensure sustained knowledge and skill improvement. We also found that residents reported increased confidence when perceiving improved residency training on this topic and strongly supported the idea of continuing education about media. We hope that our findings can lead to further studies regarding media education, implementation of media curricula among other residency programs across the nation, and, ultimately, improved trainee education regarding this pervasive, budding health issue for children and adolescents.

Appendices

A. Timeline for Curriculum.docx
B. Pretest.docx
C. Workshop 1 Slides.pptx
D. Workshop 2 Slides.pptx
E. Role-Play Patient Script.docx
F. Role-Play Physician Guide.docx
G. Role-Play Observation of Performance Checklist.docx
H. Posttest Immediately After Curriculum.docx
I. Posttest 4 Months After Curriculum.docx
J. Answer Key to Knowledge Questions.docx

All appendices are peer reviewed as integral parts of the Original Publication.

Rashi Kabra, MD, MSEd: Adolescent Medicine Fellow, Division of Adolescent Medicine, Department of Pediatrics, Cohen Children's Medical Center, Northwell Health; ORCID: https://orcid.org/0000-0002-5414-2261

Shara Steiner, DO, MACM: Associate Professor, Health Professions Education Graduate Programs, Donald and Barbara Zucker School of Medicine at Hofstra/Northwell

Jane Cerise, PhD: Associate Biostatistician, Biostatistics Unit, Feinstein Institute for Medical Research, Northwell Health

Nadia Saldanha, MD: Assistant Professor, Division of Adolescent Medicine, Department of Pediatrics, Cohen Children's Medical Center, Northwell Health

Disclosures
None to report.

Funding/Support
None to report.

Prior Presentations
Kabra R, Cerise J, Steiner S, Saldanha N. Implementation and evaluation of a novel media education curriculum for pediatric residents. Poster presented at: Society for Adolescent Health and Medicine Annual Meeting; March 7-10, 2023; Chicago, IL.

Kabra R, Cerise J, Steiner S, Saldanha N. Implementation and evaluation of a novel media education curriculum for pediatric residents. Poster presented at: Pediatric Academic Societies Annual Meeting; April 27-May 1, 2023; Washington DC.

Ethical Approval
The Northwell Health Institutional Review Board approved this project.

References

1. Committee on Public Education. Media education. *Pediatrics*. 1999;104(2):341-343. https://doi.org/10.1542/peds.104.2.341
2. Council on Communications and Media. Media use in school-aged children and adolescents. *Pediatrics*. 2016;138(5):e20162592. https://doi.org/10.1542/peds.2016-2592
3. Christakis DA, Frintner MP, Mulligan DA, Fuld GL, Olson LM. Media education in pediatric residencies: a national survey. *Acad Pediatr*. 2013;13(1):55-58. https://doi.org/10.1016/j.acap.2012.10.003
4. Rich M, Bar-on M. Child health in the information age: media education of pediatricians. *Pediatrics*. 2001;107(1):156-162. https://doi.org/10.1542/peds.107.1.156
5. Simmons M, Shalwitz J, Pollock S, Young A. *Adolescent Health Care 101: The Basics*. Adolescent Health Working Group; 2003.
6. Robertson M, Shoss MK, Broom MA. Social media: social intelligence training module. *MedEdPORTAL*. 2016;12:10442. https://doi.org/10.15766/mep_2374-8265.10442
7. Sotto-Santiago S, Sharp S, Mac J. The power of social media in the promotion and tenure of clinician educators. *MedEdPORTAL*. 2020;16:10943. https://doi.org/10.15766/mep_2374-8265.10943
8. Mi M, Stefaniak J, Solomonson W. Application of Web 2.0 technologies in enhancing teaching and learning in medical education. *MedEdPORTAL*. 2012;8:9290. https://doi.org/10.15766/mep_2374-8265.9290
9. Durning SJ, Artino AR. Situativity theory: a perspective on how participants and the environment can interact: AMEE Guide no. 52. *Med Teach*. 2011;33(3):188-199. https://doi.org/10.3109/0142159X.2011.550965
10. Family Media Plan. American Academy of Pediatrics. Accessed November 8, 2023. https://www.healthychildren.org/english/fmp/pages/mediaplan.aspx#/
11. Common Sense Media. Accessed November 8, 2023. https://www.commonsensemedia.org/

Copyright © 2023 Kabra et al. This is an open-access publication distributed under the terms of the Creative Commons Attribution license.

12. Clinician toolkit. Boston Children's Digital Wellness Lab. Accessed November 8, 2023. https://digitalwellnesslab.org/cimaid/clinician-toolkit/

13. Kirkpatrick J, Kirkpatrick WK. *An Introduction to the New World Kirkpatrick Model*. Kirkpatrick Partners; 2021. Accessed November 8, 2023. http://www.kirkpatrickpartners.com/wp-content/uploads/2021/11/Introduction-to-the-Kirkpatrick-New-World-Model.pdf

Received: April 11, 2023
Accepted: October 8, 2023
Published: December 22, 2023

Copyright © 2023 Kabra et al. This is an open-access publication distributed under the terms of the Creative Commons Attribution license.
